# Altered Expression of PDE4 Genes in Schizophrenia: Insights from a Brain and Blood Sample Meta-Analysis and iPSC-Derived Neurons

**DOI:** 10.3390/genes15050609

**Published:** 2024-05-10

**Authors:** Nitzan Burrack, Assif Yitzhaky, Liron Mizrahi, Meiyan Wang, Shani Stern, Libi Hertzberg

**Affiliations:** 1 Faculty of Health Sciences, Ben-Gurion University of the Negev, Beer-Sheva 84101, Israel; burrack@post.bgu.ac.il; 2 Clinical Research Center, Soroka University Medical Center, Beer-Sheva 84101, Israel; 3 Department of Physics of Complex Systems, Weizmann Institute of Science, Rehovot 76100, Israel; 4 Sagol Department of Neurobiology, Faculty of Natural Sciences, University of Haifa, Haifa 3103301, Israel; 5 Laboratory of Genetics, The Salk Institute for Biological Studies, La Jolla, CA 92037, USA; 6 Shalvata Mental Health Center, Affiliated with the Faculty of Medicine, Tel-Aviv University, 13 Aliat Hanoar St., Hod Hasharon 45100, Israel

**Keywords:** schizophrenia, gene expression, PDE4, meta-analysis, induced pluripotent stem cells, monozygotic twin, long term potentiation, cognitive impairment

## Abstract

Schizophrenia symptomatology includes negative symptoms and cognitive impairment. Several studies have linked schizophrenia with the PDE4 family of enzymes due to their genetic association and function in cognitive processes such as long-term potentiation. We conducted a systematic gene expression meta-analysis of four PDE4 genes (PDE4A-D) in 10 brain sample datasets (437 samples) and three blood sample datasets (300 samples). Subsequently, we measured mRNA levels in iPSC-derived hippocampal dentate gyrus neurons generated from fibroblasts of three groups: healthy controls, healthy monozygotic twins (MZ), and their MZ siblings with schizophrenia. We found downregulation of PDE4B in brain tissues, further validated by independent data of the CommonMind consortium (515 samples). Interestingly, the downregulation signal was present in a subgroup of the patients, while the others showed no differential expression or even upregulation. Notably, PDE4A, PDE4B, and PDE4D exhibited upregulation in iPSC-derived neurons compared to healthy controls, whereas in blood samples, PDE4B was found to be upregulated while PDE4A was downregulated. While the precise mechanism and direction of altered PDE4 expression necessitate further investigation, the observed multilevel differential expression across the brain, blood, and iPSC-derived neurons compellingly suggests the involvement of PDE4 genes in the pathophysiology of schizophrenia.

## 1. Introduction

Schizophrenia is one of the most debilitating mental health disorders, with a lifetime risk of approximately 0.7% [1]. The negative symptoms of schizophrenia include anhedonia, social avoidance, cognitive impairment, and lack of interest. Those are responsible for the chronic disability associated with the disease [2]. Although antipsychotic medications are the primary treatment for schizophrenia, they have limited effectiveness and abundant side effects [3]. A better understanding of the biological basis of schizophrenia is crucial for developing and introducing better treatments [4].

Schizophrenia has a highly complex genetic component etiology. Approximately 80% of the phenotype is attributed to genetics, known as heritability [5]. Genome-wide association studies (GWAS) have detected more than 250 associated loci that may predispose the development of schizophrenia [6]. However, a substantial fraction of these variants are located in regulatory rather than protein-coding regions, primarily affecting gene expression patterns [4]. For that reason, systematically studying transcriptomic data is crucial to understanding schizophrenia’s underlying pathophysiology [7].

The study of gene expression using a single cohort of brain samples has limitations. Schizophrenia exhibits a high degree of heterogeneity in terms of clinical features and the combination of genes leading to susceptibility [8]. Consequently, relevant signals can be diluted depending on the population under study. Although it has been traditionally believed that schizophrenia’s pathogenesis is localized to specific brain regions, recent evidence suggests that aspects of its pathophysiology have pan-cortical involvement [9]. The largest schizophrenia GWAS conducted to date revealed that genes exhibiting high relative expression in most regions of the human brain were enriched for schizophrenia risk variants [6]. Furthermore, this study identified an enrichment of associated variants in genes expressed specifically in neurons or involved in synaptic functioning [6]. A comprehensive gene expression study also demonstrated an enrichment of schizophrenia genetic risk in gene expression modules spanning the entire brain. These findings and the lack of regional specificity observed in structural brain imaging studies indicate that schizophrenia stems from fundamental disruptions in neuronal and synaptic functions, not confined to specific brain regions [10,11,12,13]. One way to deal with these characteristics is to integrate data from different gene expression studies, expanding multiple brain regions.

In this context, cyclic nucleotide phosphodiesterase (PDE) enzymes play an important role. They are encoded by 21 genes, grouped into 11 sub-families, and are responsible for degrading cyclic adenosine monophosphate (cAMP), a key second messenger in the cell. External signals trigger a signaling cascade within the cell by activating G-proteins. This causes an increase in cAMP levels, activating effector proteins that regulate a wide range of cellular processes [14,15]. The levels of cAMP change quickly and are highly localized in microdomains [14,16]. Proper localization and timing of the proteins involved in cAMP production and their degradation by PDEs is essential for coherent downstream signaling [17]. The PDE4 sub-family consists of four genes highly expressed in the immune and nervous systems and has been previously associated with psychiatric illnesses [18,19]. PDE4B and PDE4D are the most abundant sub-family members within the brain [19,20].

PDE4 genes were associated with long-term potentiation (LTP), which involves continuous synaptic strengthening and improves neuronal signal transmission over time [21]. The chemical synapses’ capacity to increase their strength is one of the processes at the base of neural plasticity. Memory encoding involves changes in synaptic strength. Thus, LTP is one of the central mechanisms contributing to memory and learning [21]. LTP involves local tagging of the target synapse and requires timely fashioned elevation in cAMP levels, activating several proteins via protein kinase A (PKA)-dependent phosphorylation. As LTP progresses in the CA1 region of the hippocampus, the PDE4B3 isoform oscillates, transiently modulating cAMP [22,23]. A mouse model with a mutant form of PDE4B of decreased ability to hydrolyze cAMP displayed cognitive enhancement [24]. Thus, PDE4B modulates the memory formation process and plasticity [25].

While PDE4B has been linked to LTP and neural plasticity, PDE4A was associated with synaptic gating in the prefrontal cortex (PFC) [26]. The selective gating of synaptic inputs is a physiological process at the base of working memory. PDE4A is located around the dendritic spines of the excitatory synapse. The superficial PFC is where working memory microcircuits interconnect and spine loss is detected in schizophrenia [26]. PDE4C was shown to have low expression levels in the human brain [27].

Although several PDE4B single nucleotide polymorphisms (SNPs) have been associated with schizophrenia [25,28,29,30,31] in case-control studies, two recent GWAS found no association [32,33]. In addition, a study of a Finnish population found two PDE4D variants to be highly associated with schizophrenia, possibly with a regulatory function based on their location in an intronic region within the gene. Moreover, one of these variants (rs165940) has been correlated with decreased expression of PDE4D in brain samples of healthy individuals, especially in cerebellar tissue [19]. Furthermore, a PDE4A SNP was associated with schizophrenia in a Japanese population [34]. Only a few studies examined PDE4 genes’ expression or protein levels in schizophrenia. PDE4B did not show differential expression in dorsolateral PFC (DLPFC) samples of 35 individuals with schizophrenia vs. 35 controls [31]. However, the PDE4A5 isoform has shown a trend toward decreased expression in 15 cerebellar samples of patients with schizophrenia vs. 15 healthy controls [35]. Interestingly, PDE4B2 and PDE4B4 isoform protein levels were reduced in the cerebellar tissue of 14 individuals with schizophrenia versus 15 healthy controls [28].

To explore PDE4 genes’ differential expression in schizophrenia, we conducted a systematic meta-analysis using available gene expression datasets from multiple brain regions (437 samples; 219 schizophrenia and 218 controls). We then explored whether the results were replicated in an independent cohort (515 samples; 244 schizophrenia and 271 controls) and in blood samples (300 samples; 167 schizophrenia and 133 controls). In the second stage, we measured the differential expression of PDE4 genes in hippocampal dentate gyrus (DG) neurons derived from induced pluripotent stem cells (iPSC). The hippocampus and, specifically, the dentate gyrus have been shown to be affected in schizophrenia across multiple studies [36,37,38]. These neurons, reprogrammed from fibroblasts of discordant twins for schizophrenia and controls, were used to investigate whether PDE4 genes’ differential expression occurs in earlier stages of schizophrenia.

## 2. Methods

### 2.1. Identification and Selection of Eligible Gene Expression Datasets for Meta-Analysis

**Figure 1:**
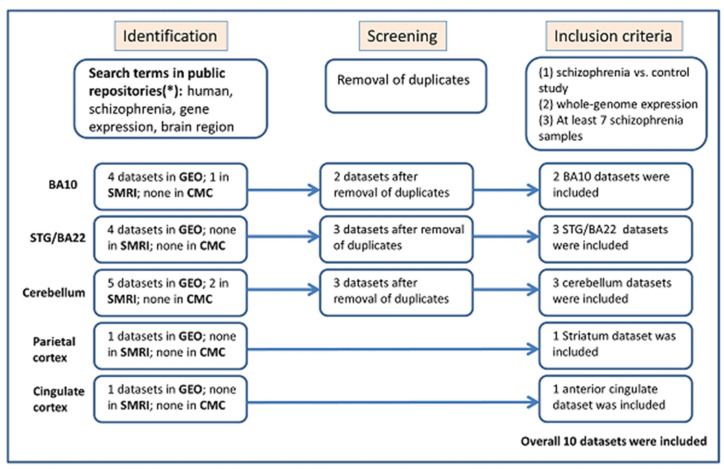
The flow of information through the different stages of the selection of gene expression datasets for meta-analysis [41]. (*) see the full description of the search performed in Section 2.1.

Publicly available gene expression datasets were searched in three public repositories: NCBI Gene Expression Omnibus (GEO) http://www.ncbi.nlm.nih.gov/geo/ (accessed on 3 February 2023), the Stanley Medical Research Institute (SMRI) Array Collection http://www.stanleyresearch.org/brain-research/array-collection/ (accessed on 1 May 2023), and the CommonMind Consortium (CMC) [39]. First, the following keywords and their combinations were used: schizophrenia, human/homo sapiens, gene expression/RNA/RNA-seq, and brain. We then searched the resulting datasets manually to include only those that meet the following inclusion criteria: human schizophrenia versus control study of postmortem Brodmann area 10, Brodmann area 22/STG, cerebellum, parietal cortex or cingulate cortex samples, a minimum of seven samples of individuals with schizophrenia, comparable conditions, and availability of gene expression preprocessed data. Figure 1 presents the workflow of eligible dataset selection, following the Preferred Reporting Items for Systematic Reviews and Meta-Analysis (PRISMA) 2020 guidelines [40].

The following information was extracted from each identified study: sample type, platform, number of cases and controls, PubMed ID, and the preprocessed gene expression data (Table 1). The overall number of samples included is 219 brain samples of individuals with schizophrenia and 218 brain samples of healthy controls. The complete dataset characteristics are described in the Supplementary Materials.

### 2.2. Statistical Analysis

For a given gene, a meta-analysis that integrates its expression in the gene expression datasets was applied as follows. The effect size (Hedges’ g [53]), the standardized difference between the expression in the disease vs. control samples, was calculated separately for each dataset. The direction of the effect size was positive if the expression in the disease group was higher than in the control group. Hedges’ g and confidence interval values were calculated for each of the datasets using the function “metacont” from the “meta” package in R, a general package for meta-analysis, version 4.9-2 [54]. The summary measure of the datasets with its confidence interval was calculated by the same function, using the random effects model [55]. To explore the effect of possible confounders, such as antipsychotic use, age, PMI, and pH of the postmortem tissue, we applied correlation analysis and linear regression analysis (see Supplementary Materials). We calculated a *p*-value for obtaining x genes with random effect Hedges lower than a given threshold. To calculate a *p*-value for obtaining x genes out of the four genes of interest, with random effect Hedges lower than a given threshold, we used a binomial distribution with the parameters: number of successes = x, n = 4, and *p* = (the number of genes with random effect Hedges < threshold)/(the overall number of genes common to at least two of the ten datasets included in the meta-analysis = 41,384). For this purpose, we calculated the random effect hedge values of all 41,384 genes.

### 2.3. iPSC-Derived Neuron Preparation and Transcription Analysis

**Figure 2:**
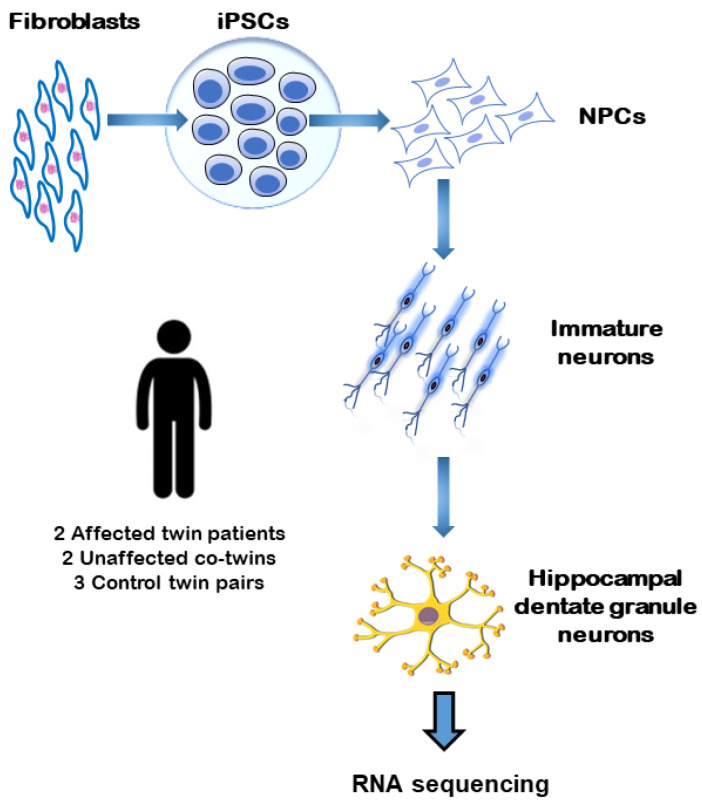
Generation of neurons from healthy twin sets and from twin sets that are discordant for schizophrenia. A schematic of the reprogramming and differentiation and the experiments performed. Abbreviations: iPSCs: induced pluripotent stem cell; NPCs: neural progenitor cells.

We examined PDE4 gene expression in vitro in iPSC-derived FACS-sorted neurons. These neurons were generated and analyzed in a study by Stern et al. [56]. The iPSCs were reprogrammed from fibroblasts of three groups: healthy controls, monozygotic twins (MZ) without a history of schizophrenia, and their MZ siblings with schizophrenia, according to DSM-IV criteria. The iPSCs were then differentiated into hippocampal neurons (DG granule neurons) and aimed to resemble the morphology and physiology of the donors’ DG neurons. The subjects’ recruitment and biopsies were conducted in Utrecht, Netherlands, at the Department of Psychiatry, UMC Utrecht. Monozygotic twin pairs, discordant for schizophrenia, and control monozygotic twin pairs from the U-TWIN cohort were asked to participate in the study [57]. The inclusion criteria are described by Stern et al. [56]. The UMC Utrecht Medical Ethical Committee approved this study and the Salk Institute obtained IRB approval. iPSCs were derived from fibroblasts for control and schizophrenia patients. To obtain mature neurons, they were first reprogrammed to form iPSC. Secondly, they were differentiated to form DG neurons, utilizing described protocols (Figure 2) [58,59]. Subsequently, we performed mRNA sequencing; to assess the significance of the differences observed between groups (Control, CO, SCZ), we performed nested ANOVA analysis [60]. The methods are elaborated in the Supplementary Materials. Altogether, fibroblast cells from five pairs of twins were successfully reprogrammed by the Salk Institute Stem Cell Core Laboratory (subject age: 32–50 years). Two pairs of subjects were discordant for schizophrenia (age at onset: 22–35 years), while three were healthy control twins (Table 2).

## 3. Results

### 3.1. PDE4B Is Downregulated in Brain Samples of Individuals with Schizophrenia

**Figure 3:**
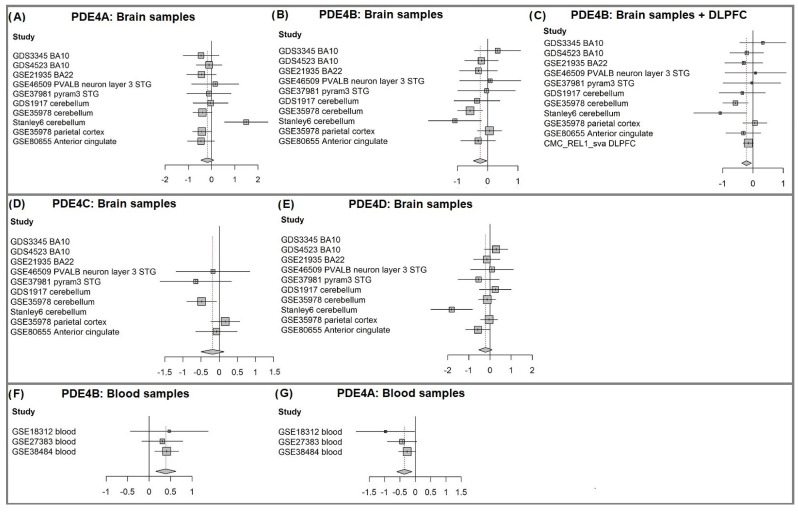
PDE4 genes meta-analysis results. (**A**) Brain sample meta-analysis of PDE4B differential expression in ten datasets of brain samples of individuals with schizophrenia vs. healthy controls. A forest plot was generated using the function “forest” from the “meta” package in R, version 4.9-2 (General Package for Meta-Analysis) [54]. Each square represents the standardized difference (Hedges’ g [53]) between schizophrenia and control for a specific dataset, with the area of the square reflecting the weight (determined by the sample size) given to that dataset in the meta-analysis. Each horizontal line represents the 95% confidence interval for the mean difference in that study. The vertical line shows the point of zero difference. The standardized difference is positive (negative) if the expression is higher (lower) in schizophrenia vs. the control group. The center of the diamond represents the overall difference across both studies and its width represents a 95% confidence interval. (**B**) Brain sample meta-analysis of PDE4B differential expression in ten brain samples datasets + the CMC DLPFC dataset of individuals with schizophrenia vs. healthy controls. (**C**) Brain sample meta-analysis of PDE4A differential expression in ten brain samples datasets of individuals with schizophrenia vs. healthy controls. (**D**) Brain sample meta-analysis of PDE4C differential expression of individuals with schizophrenia vs. healthy controls. (**E**) Brain sample meta-analysis of PDE4D differential expression of individuals with schizophrenia vs. healthy controls. (**F**) Blood sample meta-analysis of PDE4B differential expression in three datasets of blood samples of individuals with schizophrenia vs. healthy controls. (**G**) Blood sample meta-analysis of PDE4A differential expression in three datasets of blood samples of individuals with schizophrenia vs. healthy controls.

We have performed a systematic meta-analysis of the four PDE4 genes’ expression in 219 brain samples of individuals with schizophrenia vs. 218 healthy controls, integrating 10 gene expression datasets (Table 1). PDE4B was found to be downregulated in schizophrenia (Figure 3B, *p*-value = 0.029). Moreover, all four PDE4 genes show a tendency for downregulation (Figure 3A–E, Table 3; random effects Hedge value < −0.15). We next added to the meta-analysis a large dataset of dorsolateral prefrontal cortex (DLPFC) samples from the CommonMind Consortium (CMC; 251 schizophrenia patients vs. 288 controls) [39]. This further strengthened the significance of PDE4B downregulation in brain samples of patients with schizophrenia (Figure 3B, *p*-value = 0.01). 

One potential confounding factor that may influence differential expression between individuals with schizophrenia and controls is exposure to antipsychotic therapy. To assess the impact of antipsychotic use, we examined four datasets that included information on lifetime medication usage. Interestingly, no significant correlation was observed in either dataset (Figure S1, *p*-values = 0.87, 0.72, 0.1, and 0.88). Based on these results, it is unlikely that the downregulation of PDE4B is due to antipsychotic treatment. However, it is important to note that this conclusion is based on a limited number of samples for which such information was available.

To further investigate this issue, we also examined transcriptome data from the DLPFC of healthy rhesus macaques treated with clozapine, haloperidol, or placebo over six months (Table S1) [39,61]. Among the four PDE4 genes examined, only PDE4A displayed significant differential expression, specifically downregulation (*p*-value = 0.049), in the low-dose haloperidol group (0.14 mg/kg/day) compared to the placebo group. These additional analyses make it seem unlikely that antipsychotic treatment leads to PDE4 downregulation.

To account for the potential effects of age, pH, and PMI, we fitted a linear model including them as covariates for each dataset (see Section 2). The results are listed in Table S2. For PDE4B, two of the datasets showed significant downregulation in schizophrenia after accounting for age, pH, and PMI potential effects (Chen 2013, *p*-value < 0.001; Stanley#6, *p*-value = 0.03). To summarize the linear regression analysis for each of the genes, the mean t-statistic values were calculated, showing a clear tendency for downregulation for all four PDE4 genes after accounting for age, pH, and PMI effects (t-statistic = −0.42, −0.83, −1.09, and −0.43, respectively).

We note that the brain samples included in our meta-analysis are not independent as some of the datasets originate from the same brain banks (detailed in Table 1). We thus assume that a fraction of the samples originate from the same patients. To examine whether our results remain statistically significant, even when this fact is taken into account, we calculated a *p*-value for obtaining four PDE4 genes with random effects Hedges values < −0.15, using a binomial distribution (*p*-value = 0.0016; see methods). We conclude that beyond the significant downregulation of a specific gene, PDE4B, we see a significant trend toward downregulation of the four PDE4 genes as a group. This calculation used the distribution of the random effects Hedges of all genes common to the 10 datasets included in our meta-analysis (see Section 2) and thus inherently takes into account any existing dependencies between them.

### 3.2. PDE4B Downregulation Is Concentrated in a Subgroup of Patients

**Figure 4:**
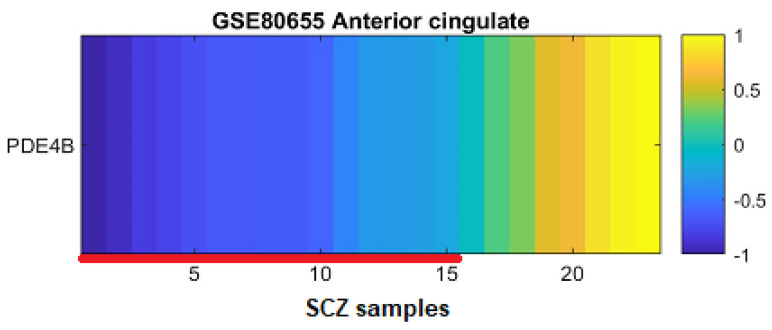
PDE4 per-sample log-fold change analysis of the anterior cingulate GSE80655 dataset. Each column represents an individual with schizophrenia. The color in entry (j) represents the Log2 fold change in the expression of PDE4B as measured in the brain sample of the individual represented in column j, Ej (Log2 (Ej/mean expression of the control samples)). Samples in which PDE4B is downregulated (bluish color) are marked with a red line along the x-axis.

In order to explore whether PDE4B downregulation is homogeneous among the patients, we performed a per-sample fold change analysis. As shown in Figure 4, the downregulation is concentrated in a subgroup of patients (marked in blue shades and marked with a red line along the x-axis). The rest of the patients (those not marked with a red line) do not show a downregulation; some even show upregulation (marked in yellow shades, Figure 4). This pattern, which suggests that the downregulation is concentrated in a subgroup of the patients, is replicated in the other 11 datasets included in our meta-analysis (Figure S2). Pearson correlation analysis between PDE4B expression levels and the sex and age of the patients did not yield statistically significant results (Figures S3 and S4; *p*-values = 0.09, 0.96, respectively).

### 3.3. Blood Samples Analysis: PDE4A Is Downregulated and PDE4B Is Upregulated in Individuals with Schizophrenia

We next explored whether PDE4A, PDE4B, PDE4C, and PDE4D are differentially expressed not only in the brain samples but also in blood samples of patients with schizophrenia and thus might serve as biomarkers. We used three publicly available schizophrenia-control blood samples datasets (overall 300 samples; 167 schizophrenia and 133 controls). Datasets characteristics are listed in Table 1. See Supplementary Materials for a detailed description of the datasets. Interestingly, PDE4B was found to be upregulated in blood samples of patients with schizophrenia (*p*-value = 0.0011), while PDE4A was found to be downregulated (*p*-value = 0.0052; Figure 3F,G). PDE4C and PDE4D did not show significant differential expression (Figure S8, *p*-values = 0.22 and 0.7, respectively).

### 3.4. PDE4 Genes Are Upregulated in Dentate Gyrus Granule Neurons Derived from Schizophrenia Patients Compared to Controls

**Figure 5:**
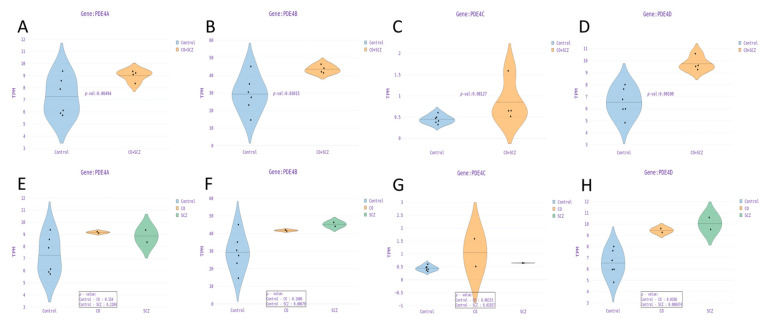
(**A**–**D**) Comparison of PDE4 gene expression levels between the control (CTR, n = 6) and the affected and unaffected monozygotic twins (CO + SCZ, n = 4). (**E**–**H**): Comparison of gene expression levels between affected patients diagnosed with schizophrenia (SCZ), their unaffected siblings (CO), and controls (CTR).

RNA sequencing was performed from RNA extracted from 7–8 week-differentiated DG neurons derived from a cohort of five twin pairs as reported in [56]. ICC confirmed that around 70% of the neurons in the cultures were PROX1 positive, indicating a high percentage of DG granule neurons [56]. A representative image is shown in Supplementary Figure S9 and a full quantification shows that over 65% of the neurons are PROX1 positive in all the lines. Two pairs were discordant to schizophrenia and three were healthy controls. We were interested in comparing the gene expression of the four PDE4 genes in the patients with schizophrenia, their unaffected twins, and the control twin pairs. For each of the affected twin lines, neurons from two different clones were grown in two separate wells (biological replicates). The biological replicate data were averaged before being used for further analysis. For the controls, two biological replicates were grown for one clone of iPSCs. Figure 5A–D presents the transcript per million (TPM) values of the four PDE4 genes in the control twins vs. the MZ twins (both the affected and their co-twin). For visualization purposes, the average TPM value of each donor’s clone samples was used but the clone factor was included in the nested ANOVA analysis. PDE4B and PDE4D were overexpressed in the MZ twins compared to the controls. At the same time, the PDE4A gene showed a trend toward overexpression (controls vs. CO + SCZ, *p* = 0.065).

PDE4B was found to be overexpressed in the affected twins, although this was marginally significant (Figure 5F, control vs. schizophrenia, *p* = 0.087; control vs. co-twin *p* = 0.16). Finally, the PDE4D was overexpressed in neurons derived from the discordant twins (Figure 5H, control vs. schizophrenia *p* = 0.01; control vs. co-twin *p* = 0.017). Similarly to our observation that the unaffected co-twin resides in an intermediate state between the controls and affected twin when considering electrophysiological recordings, similar patterns were also observed for the levels of PDE4B and PDE4D expression (Figure 5F,H) [56].

The correlation coefficients between the expression patterns of the four PDE4 genes are presented in Figure S5. We were interested in exploring other genes correlated with the expression of the PDE4 genes. The top correlated genes are shown in Figure S6A–D. Furthermore, we ran gene ontology (GO) and KEGG pathway enrichment analysis on the top genes correlated with the expression of the PDE4 genes since these may be pathways involved in the dysregulation of the PDE4 genes we detected. The results are presented in Figure S7. Supplementary Figure S7A presents the pathways for the PDE4A gene, mostly correlated with cAMP signal transduction processes. Interestingly, one of the top three pathways enriched in the list of genes correlated with PDE4B was the “synaptic vesicle cycle” (Figure S7B). Pathways of genes correlated with the PDE4C gene are presented in Figure S7E–D. No pathways were identified for PDE4D.

## 4. Discussion

In this study, we have conducted a systematic meta-analysis of four PDE4 genes’ expression in schizophrenia. We included gene expression reports from 10 datasets encompassing five different brain regions, BA10, BA22/STG, the cerebellum, the parietal cortex, and the ACC, all of which are known to be involved in the pathophysiology of the disease. Based on mRNA expression levels of 437 brain samples (219 schizophrenia and 218 controls), the results reveal significant downregulation of PDE4B in brain samples of individuals with schizophrenia compared to healthy controls. Other genes, such as PDE4A, PDE4C, and PDE4D, have shown a similar trend of decreased expression, albeit not reaching statistical significance. The analysis of the large CMC DLPFC dataset (515 samples) further validated PDE4B downregulation in schizophrenia. Notably, per-sample fold change analysis indicated that the downregulation signal is concentrated in a subgroup of the patients. In contrast, in the rest of the patients, expression levels are similar to or higher than in the controls. This finding is in concordance with the heterogeneity of the biology of schizophrenia and the inconsistency of expression patterns in previous studies. Interestingly, gene expression analysis of DG hippocampal neurons derived from iPSC showed increased expression of PDE4 genes in two individuals with schizophrenia and their co-unaffected monozygotic twins compared to three healthy control twin pairs.

Relatively few studies have previously examined PDE4B expression in schizophrenia. However, there is substantial evidence for the involvement of genetic variations of PDE4B in the pathophysiology of schizophrenia and other psychiatric disorders, emphasizing the importance of a systematic study of its differential expression in schizophrenia [25,28,29,30,31,62]. Inadequate statistical power and cohort heterogenicity may have made it difficult for other studies to detect differences in PDE4B expression [31,35]. This, along with biological heterogeneity, is likely to account for the observed inconsistencies in the direction of differential expression across datasets of the same region, such as BA10 (as illustrated in Figure 3B). Besides the downregulation of PDE4B in brain samples, PDE4A was downregulated in blood samples from schizophrenia patients. Surprisingly, PDE4B was upregulated in the blood, opposite to its differential expression in the brain tissue. Due to the importance of blood samples for detecting biomarkers, future research should focus on this direction.

The main question is whether and how PDE4B downregulation contributes to the development of schizophrenia and affects LTP and memory formation processes in a subset of the patients. Several studies employing various techniques, such as mutated PDE4B genes and pharmacological inhibition of PDE4B protein products, support this notion [24,63,64]. First, PDE4B-related SNPs were associated with schizophrenia, supporting PDE4B involvement in disease development. Moreover, expression quantitative trait loci (eQTLs) and colocalization analysis identified several schizophrenia risk variants in PDE4B that were associated with reduced PDE4B expression in both excitatory and inhibitory neurons and glial cells [65]. These findings suggest that the reduced expression of PDE4B plays a contributory role in the development of schizophrenia. Looking at the mechanism, appropriate cellular responses in LTP require a transient increase in cAMP levels. Counterintuitively, sustained elevation in cAMP levels could directly or indirectly induce cognitive and memory impairments [63,64] as demonstrated in a mice model treated chronically with Rolipram, a PDE4 inhibitor, which led to PKA upregulation and subsequent memory and learning deficits [66]. Consistent with that, mutant mice with a decreased catalytic ability of PDE4B have shown reduced contextual fear memory after seven days of fear conditioning [24]. These mice showed increased phosphorylation of the transcription factor CREB (cAMP response element binding protein), which is known to be activated when phosphorylated by PKA and promotes the transcription of genes required for late-LTP [67].

These findings support the hypothesis that PDE4B downregulation plays a causative role in schizophrenia, preceding the onset of symptoms. That being said, other evidence suggests otherwise. For instance, behavioral tests on PDE4B knock-out mice did not detect differences in fear conditioning or the acquisition and retention of spatial memory [68]. To explore the potential association between PDE4B downregulation and schizophrenia symptoms, we examined whether existing PDE4B inhibitors exert adverse effects resembling schizophrenia symptoms. We found no reported side effects resembling the negative or positive symptoms of schizophrenia. (Table S3) It should be taken into account that the decrease in PDE4B levels seen in schizophrenia patients’ brains may be a compensatory response to aberrant cAMP signaling. Factors like altered expression of different PDE4B isoforms could contribute to this signaling deficiency. Conversely, inhibiting PDE4 may paradoxically increase cAMP signaling and restore balance to the cascade, offering therapeutic benefits [24,28]. Overall, while there is support for the causal role of PDE4B downregulation in schizophrenia, preceding symptom onset, other studies present a different perspective.

In the second stage, we measured the differential expression of PDE4 genes in Hippocampal DG neurons derived from iPSC. We discovered higher mRNA levels of PDE4 A, B, and D genes neurons derived from the MZ twins (affected + co-twin) compared to the control group. However, when comparing the results of the affected and co-twins separately to the control, they were not significant, probably due to insufficient power. Being derived from donors similar in their genetic origin helps us understand the etiology of the transcriptional changes.

In the unaffected MZ siblings, PDE4B and PDE4D were expressed at intermediate levels between patients and controls. This suggests that genetic variations, supposedly identical in healthy and affected twins, may contribute to the upregulation we observed and may predispose to the condition. De novo germline mutations or epigenetic modifications might have elevated mRNA levels in the affected twins [56]. Several possible explanations explain the opposite trends in the direction of the PDE4 genes’ differential expression between postmortem brain samples and iPSC-derived neurons. First, the upregulation might represent only a subset of patients that are characterized by increased expression. It becomes increasingly acknowledged that schizophrenia is comprised different biological entities; in a recent gene expression study of DLPFC samples, two molecular subtypes of schizophrenia were identified. “Type 1” showed minimal differentially expressed genes compared to healthy controls while “Type 2” displayed several thousand differentially expressed genes [69]. Second, the MZ-derived neurons correspond to young neurons, representing transcriptional profiles at a specific disease phase [70,71,72,73]. The morphology and physiology of neurons can change along the disease course. Several studies on animal and iPSC models of brain disorders have demonstrated that neurons can have different expression and excitability patterns depending on age and the disease phase [74,75,76,77]. Third, a discrepancy between expression levels could be attributed to brain samples typically containing several types of cells, both neuronal and glial tissue [78,79]. Other studies comparing iPSC neurons and postmortem tissue transcriptomics have faced and discussed such inconsistencies [80,81,82,83].

While it is not surprising that the PDE4A-correlated genes group was enriched with cAMP binding and PDE activity pathways, it validates the mRNA levels measured and the methodology of pathway enrichment analysis of the highly correlated genes. Interestingly, PDE4B correlated genes were enriched with synaptic vesicle cycle pathway genes. This follows reports that synaptic activity is reduced in the schizophrenia neurons compared to the controls [56,84]. Moreover, these neurons had altered morphology and electrophysiological properties, as shown in [56]. Neurons derived from patients with schizophrenia had the highest membrane resistance, suggesting lower activity of ion channels, such as hyperpolarization-activated cyclic nucleotide-gated channels (HCNs), at resting membrane potentials [56]. As cAMP activates these channels, increased PDE4 expression is expected to deplete cAMP and subsequently decrease HCN1 channel activity, which could explain the increased resistance [85]. The current of the HCN channel helps keep membrane potential near the resting potential by depolarizing it. Simulated neurons exhibit hyperpolarized membrane potentials when HCN channel conductance is blocked, while in the presence of cAMP, the channels act as an excitatory factor [86]. Thus, the increased PDE4 gene expression could contribute to the hypoexcitability observed in these neurons [56].

Several aspects limit this study. Since our meta-analysis included multiple brain regions, we may not have captured transcriptional changes specific to each region. Our iPSC neurons were of the DG region, which was not included in the meta-analysis. However, research indicates that the underlying pathophysiological processes of schizophrenia and synaptic processes specifically are shared across multiple brain regions [6,9,87]. As mRNA levels mirror the neurobiological condition at the time of death, they might not represent the mechanism and expression levels when schizophrenia was first diagnosed. It is highly relevant in the case of schizophrenia, as the pathological processes are thought to start much earlier in development [88,89]. Nevertheless, we detected PDE4 genes’ differential expression in blood samples and iPSC-derived neurons, which highly support their involvement in the pathophysiology of schizophrenia. In addition, antipsychotic treatment could potentially affect gene expression. However, our correlation analysis (Figure S1) suggests that the downregulation observed in brain samples does not result from the antipsychotic treatment dose. In addition, after accounting for the potential effects of age, pH, and PMI, PDE4 genes showed a clear tendency for downregulation (Table S2). We note that the brain samples used in our meta-analysis are not independent (see shared brain banks in Table 1). While the significant downregulation of PDE4B might result from incidental overrepresentation of patients with PDE4B downregulation (by using samples from more than one brain region of these individuals), the PDE4 genes showed a trend toward downregulation as a group (*p* = 0.0016 for having four genes with random effect Hedges < −0.15), even when taking into account the use of dependent samples. The fact that we measure gene expression alone is a serious limitation, as levels of proteins do not necessarily follow trends in the expression levels of their encoding genes. However, a study detected decreased PDE4B protein levels in brain samples of individuals with schizophrenia [28]. In addition, while the sample size of the iPSC-derived neurons was small, this sample is highly unique and informative.

## 5. Conclusions

Our meta-analysis and iPSC-derived neuron experiment suggest that PDE4 genes expression is altered in schizophrenia, potentially being downregulated in a specific group of patients. Identifying and characterizing this subset of patients could aid in the identification of distinct subtypes of schizophrenia. However, this direction necessitates further study. Additionally, the analysis of blood samples from affected individuals reveals downregulation of PDE4A and upregulation of PDE4B, suggesting their potential as biomarkers for schizophrenia. The combination of evidence from multiple levels (postmortem of brain tissue, blood, iPSC-derived neurons) along with established genetic associations supports the involvement of altered PDE4 expression in the pathophysiology of schizophrenia. The opposite direction of changes in expression between postmortem and iPSC tissues warrants further investigation into PDE4 expression in schizophrenia and its biological implications.

## Figures and Tables

**Table 1:** Characteristics of individual brain and blood sample studies included in the meta-analysis. Abbreviations: SZ: schizophrenia; CNT: controls; BA: Brodmann Area; STG: superior temporal gyrus; CRBLM: cerebellum; ACC: anterior cingulate cortex; PMI: postmortem interval; M: males; F: females. Pritzker: Pritzker Neuropsychiatric Disorders Research Consortium; Pitt: Brain Tissue Donation Program at the University of Pittsburgh; MSSM; Mount Sinai School of Medicine; Penn: University of Pennsylvania; HBCC: Human Brain Collection Core within the National Institute of Mental Health’s (NIMH); SMRI: Stanley Medical Research Institute; Maryland: Maryland Brain Collection; HBTRC: The Harvard Brain Tissue Resource Center; CCHPC; Charing Cross Hospital Prospective Collection.

Brain Samples Gene Expression Studies								
Accession	Publication	Brain Region; Brain Bank	# SZ	# CNT	Platform	Mean Age (Standard Dev.)	Mean PMI (Standard Dev.)	Mean pH (Standard Dev.)
GDS3345	Iwamoto 2004 [42]	BA10; SMRI	12 7M:5F	15 9M:6F	HG U95 Av.2	SZ: 45 (14) CNT: 48 (11) *p* = 0.47	SZ: 33 (16) CNT: 24 (10) *p* = 0.07	SZ: 6.2 (0.2) CNT: 6.3 (0.2) *p* = 0.37
GDS4523	Maycox 2009 [43]	BA10; CCHPC	27 19M:8F	23 12M:11F	HG U133 Plus 2.0	SZ: 73 (15) CNT: 69 (22) *p* = 0.45	SZ: 8.2 (7) CNT: 10 (4) *p* = 0.3	SZ:6.1 (0.2) CNT: 6.5 (0.3) *p* = 8 × 10^−6^
GSE21935	Barnes 2011 [44]	BA22; CCHPC	23 13M:10F	17 9M:8F	U133 Plus 2.0 Array	SZ: 72 (17) CNT: 65 (22) *p* = 0.25	SZ: 7 (6) CNT: 9 (4) *p* = 0.36	SZ: 6.2 (0.2) CNT: 6.5 (0.3) *p* = 2.3 × 10^−6^
GSE37981	Pietersen 2014 pyramidal [45]	STG; HBTRC	9 4M:5F	8 4M:4F	U133 X3P Array	SZ: 67 (20) CNT: 67 (21) *p* = 0.99	SZ: 17 (5) CNT: 18 (3) *p* = 0.71	Not provided
GSE46509	Pietersen 2014 parvalbumin [46]	STG; HBTRC	7 3M:4F	8 4M:4F	U133 X3P Array	SZ: 69 (22) CNT: 67 (21) *p* = 0.87	SZ: 15.8 (6) CNT: 18 (3) *p* = 0.38	Not provided
GDS1917	Paz 2006 [47]	CRBLM; Maryland	13 13M:0F	14 14M:0F	U133 Plus 2.0 Array	SZ: 46 (12) CNT: 43 (10) *p* = 0.5	SZ: 12.8 (5) CNT: 15.6 (6) *p* = 0.18	Not provided
GSE35978	Chen 2013 [48]	CRBLM; SMRI	44 32M:12F	50 31M:19F	Gene 1.0 ST Array	SZ: 43 (9); CNT: 46 (9) *p* = 0.18	SZ: 33 (15) CNT: 28 (11) *p* = 0.042	SZ: 6.4 (0.2) CNT: 6.5 (0.3) *p* = 0.44
Stanley#6		CRBLM; SMRI	10 7M:3F	14 9M:5F	U95 Av2 Array	SZ: 46 (14); CNT: 47 (9) *p* = 0.83	SZ: 34 (14) CNT: 24 (10) *p* = 0.071	SZ: 6.2 (0.2) CNT: 6.3 (0.2) *p* = 0.82
GSE35978	Chen 2013 [48]	Parietal cortex; SMRI	51 37M:14F	45 31M:14F	Gene 1.0 ST Array	SZ: 43 (10); CNT: 46 (9) *p* = 0.14	SZ: 31 (16) CNT: 27 (12) *p* = 0.17	SZ: 6.4 (0.3) CNT: 6.5 (0.3) *p* = 0.015
GSE80655	Ramaker 2017 [49]	ACC; Pritzker	23 20M:3F	24 21M:3F	Illumina HiSeq 2000	SZ: 43 (9); CNT: 50 (13) *p* = 0.043	SZ: 21 (9) CNT: 22 (7) *p* = 0.62	SZ: 6.8 (0.2) CNT: 6.9 (0.1) *p* = 0.044
			Overall: 219 155M:64F	Overall: 218 144M:74F		SZ: 52.1 (18) CNT: 51.8 (16) *p* = 0.85	SZ: 23.5 (16) CNT: 21.6 (11) *p* = 0.15	SZ: 6.4 (0.3) CNT: 6.5 (0.3) *p* = 5 × 10^−7^
CMC Release 1	Hoffman 2019 [39]	DLPFC; MSSM Penn Pitt HBCC	244 154M:90F	271 157M:114F	Illumina HiSeq 2500	SZ: 69 (17) CNT: 65 (19) *p* = 0.012	SZ: 20 (13) CNT: 14 (8) *p* = −10^12^	SZ: 6.5 (0.3) CNT: 6.6 (0.3) *p* = 0.003
**Blood Sample Gene Expression Studies**								
**Accession**	**Publication**	**Blood Samples Type**		**# SZ**		**# CNT**	**Platform**	**Mean Age (Standard Dev.)**
GSE18312	Bousman 2010 [50]	Whole blood		12 9M:3F		8 5M:3F	HuEx-1_0-st	SZ: 43 (9); CNT: 45 (7) *p* = 0.57
GSE27383	Van Beveren 2012 [51]	Peripheral Blood Mononuclear Cells (PBMCs)		41 41M:0F		29 29M:0F	HG-U133_Plus_2	Not provided
GSE38484	De Jong 2012 [52]	Whole blood		104 75M:29F		96 42M:54F	Illumina HumanHT-12 V3.0	SZ: 40 (11); CNT: 39 (14) *p* = 0.85
				Overall: 167		Overall: 133		

**Table 2:** A table describing the cohort of three groups: healthy controls, monozygotic twins (MZ) without a history of schizophrenia, and their MZ siblings with schizophrenia, according to DSM-IV criteria.

Twin Type	Type	Sex	Diagnosis DSM IV	Age	Age Onset	Sex
MZ	Patient	M	Paranoid schizophrenia	46	22	M
MZ	Co-twin	M	Depressive Disorder NOS	46	24	M
MZ	Patient	F	Paranoid schizophrenia	50	35	F
MZ	Co-twin	F	Major Depressive Disorder, Recurrent, In Partial Remission	50	25	F
MZ	Control	M	No diagnosis or condition on Axis I or Axis 2	35	-	M
MZ	Control	M		35	-	M
MZ	Control	F		46	-	F
MZ	Control	F		46	-	F
MZ	Control	F		32	-	F
MZ	Control	F		32	-	F

**Table 3:** PDE4 gene meta-analysis results. The standardized difference (random effects Hedges [53]) is negative (red) if the expression is lower in schizophrenia vs. the control group. The color intensity is proportional to the random effects Hedges value. A gene is defined as significantly down- or upregulated if its confidence interval does not cross zero. The lower and upper 95% confidence interval limits are given in the third and fourth columns. Statistically significant findings appear in bold.

Gene Symbol	Random Effects Hedges	Lower	Upper
**PDE4A**	**−0.18**	−0.46	0.1
**PDE4B**	**−0.25**	−0.47	−0.03
**PDE4C**	**−0.18**	−0.5	0.13
**PDE4D**	**−0.21**	−0.53	0.1

## Data Availability

The data used for the Meta-Analysis were obtained from publicly available repositories, as described in the methods section and Supplementary Materials of the paper. For inquiries regarding data obtained from the iPSC neurons, please get in touch with Dr. Shani Stern at sstern@univ.haifa.ac.il.

## Supplementary Materials

The following supporting information can be downloaded at https://www.mdpi.com/article/10.3390/genes15050609/s1. Figure S1. Pearson Correlation between antipsychotic treatment and PDE4B expression levels; Figure S2. Per-sample Log fold change analysis. Each subplot represents one dataset; Figure S3. Pearson Correlation between PDE4B Log2 expression and sex; Figure S4. Pearson Correlation between PDE4B Log2 expression and the age of the patients with schizophrenia in the 11 brain samples datasets included in the meta-analysis; Figure S5. Inter-gene correlation between PDE4 genes; Figure S6. Correlation of other genes with PDE4 genes; Figure S7. Pathway analysis for PDE4 correlated genes; Figure S8. Meta-analysis of PDE4C and PDE4D differential expression in three datasets of blood samples of individuals with schizophrenia vs. healthy controls; Figure S9. Immunocytochemistry (ICC) for Prox1, NeuN, Map2, DAPI in DG neurons; Table S1. Differential expression statistics of DLPFC of healthy rhesus macaques treated with Haloperidol low dosage vs. placebo; Table S2. Linear models using age, pH and PMI as covariates t-statistic values and associated *p*-values for each of the brain samples datasets for PDE4 genes; Table S3. Pharmacological agents with interaction with PDE4B and their related properties. References [90,91,92] are cited in the supplementary materials.
