# Supplementary material for: Altered Expression of PDE4 Genes in Schizophrenia: Insights from a Brain and Blood Sample Meta-Analysis and iPSC-Derived Neurons
Source: Genes (Basel). 2024 May 10;15(5):609. doi: 10.3390/genes15050609 (PMC11121586; doi:10.3390/genes15050609)
Supplement: Supplementary file 1 [file genes-15-00609-s001.zip › genes-2461057-supplementary.pdf]

SUPPLEMENTARY INFORMATION

Altered Expression of PDE4 Genes in Schizophrenia: Insights from a Brain and Blood Sample Meta-Analysis and iPSC-Derived Neurons

Nitzan Burrack [1,2], Assif Yitzhaky [3], Liron Mizrahi [4], Meiyan Wang [5], Shani Stern [4,*,†] and Libi Hertzberg [3,6,*,†]

[1] Faculty of Health Sciences, Ben-Gurion University of the Negev, Beer-Sheva 84101, Israel; burrack@post.bgu.ac.il
[2] Clinical Research Center, Soroka University Medical Center, Beer-Sheva 84101, Israel
[3] Department of Physics of Complex Systems, Weizmann Institute of Science, Rehovot 76100, Israel; assif.yitzhaky@weizmann.ac.il
[4] Sagol Department of Neurobiology, Faculty of Natural Sciences, University of Haifa, Haifa 3103301, Israel; liron.mizrahi@gmail.com
[5] Laboratory of Genetics, The Salk Institute for Biological Studies, La Jolla, CA 92037, USA; mwang@salk.edu
[6] Shalvata Mental Health Center, Affiliated with the Faculty of Medicine, Tel-Aviv University, 13 Aliat Hanoar St., Hod Hasharon 45100, Israel
* Correspondence: sstern@univ.haifa.ac.il (S.S.); libi.hertzberg@gmail.com (L.H.); Tel.: +972-4-6647934 (S.S.); +972-9-7478626 (L.H.); Fax: +972-9-7478688 (L.H.)
† These authors contributed equally to this work.

Contents

Supplementary Methods..3

Brain samples datasets characteristics *3*
Iwamoto 2004 BA10 dataset 3
Maycox 2009 BA10 dataset 3
Barnes 2011 BA22 dataset 4
Pietersen 2014 Pyramidal STG dataset 4
Pietersen 2014 Parvalbumin STG dataset 5
Paz 2006 cerebellum dataset 5
Chen 2013 cerebellum dataset 5
Stanley study #6 cerebellum dataset 6
Chen 2013 parietal cortex dataset 6
Ramaker 2017 anterior cingulate gyrus (AnCg) dataset 6
CMC Dorsolateral Prefrontal Cortex (DLPFC) dataset (release 1) 7

Blood samples datasets characteristics *7*
Bousman et al. 2010, GEO accession: GSE18312 7
Van Beveren et al. 2012, GEO accession: GSE27383 7
De Jong et al. 2012, GEO accession: GSE38484 8
Examination of potential confounding factors 8

iPSC derived neurons *8*
iPSC reprogramming and neuron differentiation 8
RNA sequencing analysis 9

Supplementary Results 9

Association between drugs' adverse effects and symptoms resembling schizophrenia *17*

LIST OF FIGURES

Figure S1. Pearson Correlation between antipsychotic treatment and PDE4B expression levels.......... 10
Figure S2. Per-sample Log fold change analysis. Each subplot represents one dataset. 11
Figure S3. Pearson Correlation between PDE4B Log2 expression and sex. 12
Figure S4. Pearson Correlation between PDE4B Log2 expression and the age of the patients with schizophrenia in the 11 brain samples datasets included in the meta-analysis. 13
Figure S5. Inter-gene correlation between PDE4 genes.......... 14
Figure S6. Correlation of other genes with PDE4 genes. 14
Figure S7. Pathway analysis for PDE4 correlated genes. 15
Figure S8. Meta-analysis of PDE4C and PDE4D differential expression in three datasets of blood samples of individuals with schizophrenia vs. healthy controls.......... 15
Figure S9. Immunocytochemistry (ICC) for Prox1, NeuN, Map2, DAPI in DG neurons.......... 16

LIST OF TABLES

Table S1. Differential expression statistics of DLPFC of healthy rhesus macaques treated with Haloperidol low dosage vs. placebo 9
Table S2. Linear model using age, pH and PMI as covariates t-statistic values and associated p-values for each of the brain samples datasets for PDE4 genes.. 10
Table S3. Pharmacological agents with interaction with PDE4B and their related properties. 17

Supplementary Methods

Brain samples datasets characteristics

Iwamoto 2004 BA10 dataset

The dataset GDS3345 [42] was downloaded from the GEO database (https://www.ncbi.nlm.nih.gov/sites/GDSbrowser?acc=GDS3345). The initial dataset consisted of 50 prefrontal cortex (BA10) brain samples, including subjects with SCZ (n=13) and controls (n=15). See Figure 2S for samples' characteristics. Samples were run on Affymetrix Human Genome U95 Version 2 Arrays. **Normalization method:** As described in GSE12654_series_matrix.txt (available at https://www.ncbi.nlm.nih.gov/geo/query/acc.cgi?acc=GSE12654), "Hybridization signal on the chip was scanned using an HP GeneArray scanner (Hewlett-Packard). The data were normalized by median centering by GeneSpring software (Agilent)". We then applied threshold and log2." The threshold was determined using scatter plots of normal samples in order to estimate the noise level (the threshold after log2 that was used is 0). **Outlier removal:** Outlier samples removal was applied similarly to [90] and as described above. One schizophrenia sample was removed. After outlier removal, 27 samples were left (12 schizophrenia and 15 controls). **Filtering:** The initial number of probe-sets was 12,558. 1) In case of a gene symbol with multiple probe-sets, the probe-set with the highest mean expression over the samples was taken into account and the other probe-sets were discarded. Number of genes after this step: 9,467. 2) Genes that are absent (values equal or lower than the threshold) in more than 70% of both the schizophrenia and the control samples, were filtered out. Number of genes after filtering: 5,848.

Maycox 2009 BA10 dataset

The dataset GDS4523 [43] was downloaded from the GEO database (https://www.ncbi.nlm.nih.gov/sites/GDSbrowser?acc=GDS4523). The dataset consists of 51 BA10 brain samples from subjects with schizophrenia (n=28) and healthy controls (n=23). See Figure 2 for samples' characteristics. Samples were run on Affymetrix Human Genome U133 Plus 2.0 Arrays. **Normalization method:** Arrays were scanned on a GeneChip Scanner 3000 and fluorescence intensity for each feature of the array was obtained by using GeneChip Operating Software (Affymetrix) [43]. As described in GSE17612_series_matrix.txt (available at https://www.ncbi.nlm.nih.gov/geo/query/acc.cgi?acc=GSE17612), the data were analyzed with Microarray Suite version 5.0 (MAS 5.0) using Affymetrix default analysis settings and global scaling as normalization method. The trimmed mean target intensity of each array was set to 150. We then applied threshold and log2. The threshold value was determined using scatter plots of healthy control samples in order to estimate the noise level (the threshold after log2 that was used is 4). **Outlier removal:** Outlier samples removal was applied similarly to [90]. Shortly, the following steps were applied: 1) Pearson correlation coefficient values between each pair of samples were calculated. 2) For each sample, the mean correlation with all other sample was calculated. 3) The mean, M, and standard deviation, S, of all samples' mean values (calculated in 2.) were calculated. 4) Samples whose mean correlation (found in 2.) were smaller than M-3*S were considered as outliers and were removed from the rest of the analysis. One schizophrenia sample was removed. After outlier removal, 50 samples were left (27 schizophrenia and 23 controls). **Filtering**: The initial number of probe-sets was 54,613. 1) In case of a gene symbol with multiple probe-sets, the probe-set with the highest mean expression over the samples was taken into account and the other probe-sets were discarded. Number of genes after this step: 30,805. 2) Genes that were absent (values equal or lower than the

threshold) in more than 70% of both the schizophrenia and the control samples, were filtered out. Number of genes after filtering: 27,261.

Barnes 2011 BA22 dataset

The dataset GSE21935 [44] was downloaded from https://www.ncbi.nlm.nih.gov/geo/query/acc.cgi?acc=GSE21935. The initial dataset consists of 42 superior temporal cortex (BA22) brain samples from subjects with schizophrenia (n=23) and healthy controls (n=19). Samples were run on Affymetrix Human Genome U133 Plus 2.0 Arrays. **Normalization method:** Arrays were scanned on a GeneChip Scanner 3000, and fluorescence intensity was obtained by using GeneChip Operating Software [44]. "The data were analyzed with Microarray Suite version 5.0 (MAS 5.0) using Affymetrix default analysis settings and global scaling as normalization method. As described in GSE21935_series_matrix.txt (available at https://www.ncbi.nlm.nih.gov/geo/query/acc.cgi?acc=GSE21935), the trimmed mean target intensity of each array was arbitrarily set to 150". We then applied threshold and log2. The threshold was determined using scatter plots of healthy control samples in order to estimate the noise level (the threshold after log2 that was used is 4). **Outlier removal:** Outlier samples removal was applied similarly to [90]. Shortly, the following steps were applied: 1) Pearson correlation coefficient values between each pair of samples were calculated. 2) For each sample, the mean correlation with all other sample was calculated. 3) The mean, M, and standard deviation, S, of all samples' mean values (calculated in 2.) were calculated. 4) Samples whose mean correlation (found in 2.) were smaller than M-3*S were considered as outliers and were removed from the rest of the analysis. After the removal, the dataset consists of 40 samples (23 schizophrenia and 17 controls). **Filtering:** Initial number of probe-sets: 54,675 (45,772 with gene symbols). 1) In case of gene symbol with multiple probes – the probe with the highest mean over the samples was taken into account and the other probes were discarded. Number of genes after this step: 22,880. 2) Genes that are absent (values equal or lower than the threshold) in more than 70% of the schizophrenia and the control samples, were filtered out. Number of genes after filtering: 17,376. Comment: The samples passed quality control [44].

Pietersen 2014 Pyramidal STG dataset

The dataset GSE37981 [45] was downloaded from the GEO database (https://www.ncbi.nlm.nih.gov/geo/query/acc.cgi?acc=GSE37981). The initial dataset consists of 18 brain samples isolated from pyramidal cells in layer III of the superior temporal gyrus (STG) with schizophrenia (n=9) and healthy controls (n=9). The samples were run on Affymetrix Human X3P Array. **Normalization method:** "Each array was scanned twice and the Affymetrix Microarray Suite 5.1 software averaged the two images to compute an intensity value for each probe cell within each probe set" [45].

We then applied threshold and log2. The threshold value was determined using scatter plots of healthy control samples in order to estimate the noise level (the threshold after log2 that was used is 3). **Outlier removal:** Outlier samples removal was applied similarly to [90] and as described above. After outlier removal, 1 normal sample was removed. After the removal, the dataset consists of 17 samples (9 schizophrenia and 8 controls). **Filtering**: Initial number of probe-sets: 61,359 (48,860 with gene symbols). 1) In case of a gene symbol with multiple probe-sets, the probe-set with the highest mean expression over the samples was taken into account and the other probe-sets were discarded. Number of genes after this step: 21,606. 2) Genes that were absent (values equal or lower than the threshold) in more than 70% of both the schizophrenia and the control samples, were filtered out. Number of genes after filtering: 17,886.

Pietersen 2014 Parvalbumin STG dataset

The dataset GSE46509 [46] was downloaded from the GEO database (https://www.ncbi.nlm.nih.gov/geo/query/acc.cgi?acc=GSE46509). The initial dataset consists of 16 brain samples isolated from parvalbumin cells in layer 3 of the superior temporal gyrus (STG) with schizophrenia (n=8) and healthy controls (n=8). The samples were run on Affymetrix Human X3P Array. **Normalization method:** As described in GSE46509_series_matrix.txt (available at https://www.ncbi.nlm.nih.gov/geo/query/acc.cgi?acc=GSE46509), "the scanning procedures were performed with a GeneChip® Model 3000 7G scanner using the Affymetrix GCOS v1.3 operating system". We then applied threshold and log2. The threshold value was determined using scatter plots of healthy control samples in order to estimate the noise level (the threshold after log2 that was used is 3). **Outlier removal:** Outlier samples removal was applied similarly to [90] and as described above. One schizophrenia sample was removed. After the removal, the dataset consists of 15 samples (7 schizophrenia and 8 controls). **Filtering**: Initial number of probe-sets: 61,359 (48,860 with gene symbols). 1) In case of a gene symbol with multiple probe-sets, the probe-set with the highest mean expression over the samples was taken into account and the other probe-sets were discarded. Number of genes after this step: 21,606.
2) Genes that were absent (values equal to or lower than the threshold) in more than 70% of both the schizophrenia and the control samples, were filtered out. Number of genes after filtering: 17,876

Paz 2006 cerebellum dataset

The dataset GDS1917 [47] was downloaded from the GEO database (https://www.ncbi.nlm.nih.gov/sites/GDSbrowser?acc=GDS1917). The initial dataset consists of 28 brain samples corresponding to the crus I/VIIa area of the cerebellum from subjects with schizophrenia (n=14) and healthy controls (n=14) (The samples were obtained from the Maryland Brain Collection). The samples were run on Affymetrix Human Genome U133 Plus 2.0 Array. **Normalization method:** We applied threshold and Log2 transformation. The threshold value was determined using scatter plots of healthy control samples in order to estimate the noise level (the threshold after Log2 that was used is 4). **Outlier removal:** Outlier samples removal was applied similarly to [90]. Shortly, the following steps were applied: 1) Pearson correlation coefficient values between each pair of samples were calculated. 2) For each sample, the mean correlation with all other sample was calculated. 3) The mean, M, and standard deviation, S, of all samples' mean values (calculated in 2.) were calculated. 4) Samples whose mean correlation (found in 2.) were smaller than M-3*S were considered as outliers and were removed from the rest of the analysis. One schizophrenia sample was removed. After the removal, 27 samples were left (13 schizophrenia and 14 controls). **Filtering**: The initial number of probe-sets was 54,613. 1) In case of a gene symbol with multiple probe-sets, the probe-set with the highest mean expression over the samples was taken into account and the other probe-sets were discarded. Number of genes after this step: 30,803. 2) Genes that were absent (values equal or lower than the threshold) in more than 70% of both the schizophrenia and the control samples, were filtered out. Number of genes after filtering: 27,329.

Chen 2013 cerebellum dataset

The dataset GSE35978 [48] was downloaded from the GEO database (https://www.ncbi.nlm.nih.gov/geo/query/acc.cgi?acc=GSE35978). The initial dataset consisted of 312 brain samples, including subjects with schizophrenia (n=44) and healthy controls (n=50) from the cerebellum. Samples were run on Affymetrix Human Gene 1.0 ST Array [transcript (gene) version]. **Normalization method:** "The data were analyzed by RMA using Affymetrix Expression Console with default analysis settings" (described in GSE35978_series_matrix.txt, available at the aforementioned link).

After applying RMA, no threshold or log2 are needed. That is because the RMA normalization method includes the following steps: 1. Background correction 2. Quantile normalization 3. Log2 transformation. **Outlier removal:** Outlier samples removal was applied similarly to [90] and as described above. After outlier removal no sample was removed. **Filtering**: The initial number of probe-sets was 33,297 (25,293 with gene symbols). In case of a gene symbol with multiple probe-sets, the probe-set with the highest mean expression over the samples was taken into account and the other probe-sets were discarded. Number of genes after this step: 23,307.

Stanley study #6 cerebellum dataset

The Stanley dataset ID 6 (Investigator: Feinberg) was downloaded from The Stanley Online Genomics Database (https://www.stanleygenomics.org/). The dataset consists of 25 cerebellum samples from subjects with schizophrenia (n=11) and healthy controls (n=14). **Array type:** Affymetrix hgu95av2. **Normalization method:** RMA (Robust Multiarray Average). After applying RMA, no threshold or log2 are needed. That is because the RMA normalization method includes the following steps: 1. Background correction 2. Quantile normalization 3. Log2 transformation. **Outlier removal:** Outlier samples removal was applied similarly to [90] and as described above. One schizophrenia sample was removed. After the removal, the dataset consists of 10 schizophrenia samples and 14 healthy control samples. **Filtering**: The initial number of probe-sets was 12,159. In case of a gene symbol with multiple probe-sets, the probe-set with the highest mean expression over the samples was taken into account and the other probe-sets were discarded. Number of genes after this step: 9,081.

Chen 2013 parietal cortex dataset

The dataset GSE35978 [48] was downloaded from the GEO database (https://www.ncbi.nlm.nih.gov/geo/query/acc.cgi?acc=GSE35978). The initial dataset consisted of 312 brain samples, including subjects with schizophrenia (n=51) and healthy controls (n=50) from the parietal cortex. Samples were run on Affymetrix Human Gene 1.0 ST Array [transcript (gene) version]. **Normalization method:** "The data were analyzed by RMA using Affymetrix Expression Console with default analysis settings" (described in GSE35978_series_matrix.txt, available at the aforementioned link). After applying RMA, no threshold or log2 are needed. That is because the RMA normalization method includes the following steps: 1. Background correction 2. Quantile normalization 3. Log2 transformation. **Outlier removal:** Outlier samples removal was applied similarly to [90] and as described above. (**Note**: this procedure was performed on the initial dataset of 312 samples). 5 control samples were removed. After the removal, the dataset consists of 51 schizophrenia samples and 45 healthy control samples. **Filtering**: The initial number of probe-sets was 33,297 (25,293 with gene symbols). In the case of a gene symbol with multiple probe-sets, the probe-set with the highest mean expression over the samples (of both brain regions) was taken into account and the other probe-sets were discarded. Number of genes after this step: 23,307.

Ramaker 2017 anterior cingulate gyrus (AnCg) dataset

The dataset GSE80655 [49] was downloaded from the GEO database (https://www.ncbi.nlm.nih.gov/geo/query/acc.cgi?acc=GSE80655). The initial dataset consisted of 281 brain samples, including subjects with schizophrenia (n=24) and healthy controls (n=24) from the anterior cingulate gyrus (AnCg). Samples were run on Illumina HiSeq 2000 (Homo sapiens). **Normalization method:** "RNA-seq paired-end FASTQ files were aligned to hg19 using Tophat and Cufflinks. Read counts for each transcript were compiled across all post-mortem tissues samples" (described in GSE80655_series_matrix.txt, available at the aforementioned link). We then applied threshold and log2. The threshold value was determined using scatter plots of healthy control samples in order to estimate the

noise level (the threshold after log2 that was used is 3). **Outlier removal:** Outlier samples removal was applied similarly to [90] and as described above. (**Note:** this procedure was performed on the initial dataset of 281 samples). 1 schizophrenia sample was removed. After the removal, the dataset consists of 23 schizophrenia samples and 24 healthy control samples. **Filtering**: Initial number of probe-sets: 57,905 (52,442 with gene symbols). 1) In the case of a gene symbol with multiple probe-sets, the probe-set with the highest mean expression over the samples (of both brain regions) was taken into account and the other probe-sets were discarded. Number of genes after this step: 51,310. 2) Genes that were absent (values equal or lower than the threshold) in more than 70% of both the schizophrenia and the control samples, were filtered out. Number of genes after filtering: 23,175.

CMC Dorsolateral Prefrontal Cortex (DLPFC) dataset (release 1)

The CommonMind Consortium (CMC) dataset [39] (release 1) was downloaded from the CommonMind Consortium Knowledge Portal (https://www.synapse.org/#!Synapse:syn5609491). The dataset consists of 585 (after omitting 8 samples whose clinical data were absent) dorsolateral prefrontal cortex (DLPFC) samples including subjects with schizophrenia (n=253) and healthy controls (n=277). Samples were run on Illumina HiSeq 2500. Normalization method: surrogate variable analysis (SVA), excluding SNP-inferred ancestry components []. Outlier removal: Outlier samples removal was applied similarly to [90] and as described above. (Note: this procedure was performed on the initial dataset of 585samples). 9 schizophrenia and 6 control samples were removed. After the removal, the dataset consists of 244 schizophrenia samples and 271 healthy control samples. Filtering: The initial number of ENSEMBL Gene ID is 16,423. After omitting ENSEMBL IDs without gene symbols, 15,948 unique gene symbols are left.

Blood samples datasets characteristics

Bousman et al. 2010, GEO accession: GSE18312

The dataset GSE18312 [50] was downloaded from the GEO database (https://www.ncbi.nlm.nih.gov/geo/query/acc.cgi?acc=GSE18312). The original dataset consisted of 30 whole blood samples, including subjects with schizophrenia (n=13) and healthy controls (n=8). **Platform:** Affymetrix Human Exon 1.0 ST Array [transcript (gene) version]. **Normalization method:** "The data were analyzed with Partek Genomics Suite v6.4 using RMA to analyze probe intensities and to determine differential gene expression between diagnostic and control groups" (described in GSE18312_series_matrix.txt, available at the aforementioned link). **Outlier removal:** Outlier samples removal was applied similarly to [90] and as described above. (**Note**: this procedure was performed on the initial dataset of 30 samples). 1 schizophrenia sample was removed. After the removal, the dataset consists of 12 schizophrenia samples and 8 healthy control samples. **Filtering**: The initial number of gene symbols was 17,634. In case of a gene symbol with multiple probe-sets, the probe-set with the highest mean expression over the samples was taken into account and the other probe-sets were discarded. Number of genes after this step: 17,324.

Van Beveren et al. 2012, GEO accession: GSE27383

The dataset GSE27383 [51] was downloaded from the GEO database (https://www.ncbi.nlm.nih.gov/geo/query/acc.cgi?acc=GSE27383). The dataset consists of 72 Peripheral Blood Mononuclear Cells (PBMCs) samples from subjects with schizophrenia (n=43) and healthy controls (n=29). Samples were run on Affymetrix Human Genome U133 Plus 2.0 Arrays. **Normalization method:** GeneChips were scanned using the Hewlett-Packard GeneArray Scanner G2500A. Raw intensity values of all samples were normalized by RMA normalization (Robust Multichip Analysis) (background correction

and quantile normalization) using Partek version 6.4 (described in GSE27383_series_matrix.txt, available at the aforementioned link). **Outlier removal:** Outlier samples removal was applied similarly to [90] and as described above. 2 schizophrenia samples were removed. After the removal, the dataset consists of 41 schizophrenia samples and 29 healthy control samples. **Filtering**: The initial number of probe-sets was 54,613 (45,772 with gene symbols). In case of a gene symbol with multiple probe-sets, the probe-set with the highest mean expression over the samples was taken into account and the other probe-sets were discarded. Number of genes after this step: 22,880.

De Jong et al. 2012, GEO accession: GSE38484

The dataset GSE38484 [52] was downloaded from the GEO database (https://www.ncbi.nlm.nih.gov/geo/query/acc.cgi?acc=GSE38484). The dataset consists of 202 whole blood samples from subjects with schizophrenia (n=106) and healthy controls (n=96). Samples were run on Illumina HumanHT-12 V3.0 expression beadchip. **Normalization method:** Data were background corrected in Beadstudio (v. 3.2.3) and transformed (vst) and normalized (rsn) in Lumi (R package) (described in GSE38484_series_matrix.txt, available at the aforementioned link). **Outlier removal:** Outlier samples removal was applied similarly to [90] and as described above. Two schizophrenia samples were removed. After the removal, the dataset consists of 104 schizophrenia samples and 96 healthy control samples. **Filtering**: The initial number of probe-sets was 48,743 (36,108 with gene symbols). In case of a gene symbol with multiple probe-sets, the probe-set with the highest mean expression over the samples was taken into account and the other probe-sets were discarded. Number of genes after this step: 25,142.

Examination of potential confounding factors

To explore a potential association between differential expression and antipsychotic medications, we performed correlation analyses between gene expression pattern and Fluphenazine equivalent dosage, using the datasets for which this information was available. Pearson Correlation between lifetime quantity of Fluphenazine or equivalent antipsychotic (in mg) and gene expression was calculated, along the individuals with schizophrenia.

In order to account for the potential effects of the age of the patients, brain samples pH and postmortem interval (PMI), a multiple least squares regression model was fitted to each gene[91], using the MATLAB function “fitlm” with default parameters. The diagnosis coefficient was statistically tested for being nonzero, implying an effect for schizophrenia beyond any other effect of the covariates. This produced a t-statistic and a corresponding p-value. To summarize the linear regression analysis, the mean t-statistic values were calculated.

iPSC derived neurons

iPSC reprogramming and neuron differentiation

iPSCs were derived from fibroblasts for control and schizophrenia patients using the Cyto-Tune Sendai reprogramming kit (Invitrogen) according to the manufacturer’s instructions. iPSCs were characterized for a normal karyotype as well as for pluripotency. Embryoid bodies (Ebs) were formed by dissociation of iPSC colonies using dispase and then plated in ultra-low attachment plates. The mTeSR was replaced on the following day with DMEM/F12 supplemented with N2 and B27. For floating Ebs were treated with DKK1, SB43154, noggin, and cyclopamine for 20 days. Ebs were then plated onto poly-L-ornithine/laminin-coated plates. The rosettes were manually collected based on their morphology and dissociated with accutase after one week and plated onto poly-L-

ornithine/laminin-coated plates in NPC media containing DMEM/F12, N2, B27, laminin, and FGF2. To obtain mature neurons, NPCs were differentiated in DMEM/F12 supplemented with N2, B27, BDNF, dibutyrl-cyclicAMP, ascorbic acid (Sigma), laminin, and Wnt3a for three weeks. Wnt3a was removed after 3 weeks. Neurons were infected with the Prox1::eGFP lentiviral vector and neurons were sorted before RNA preparation based on their GFP expression.

RNA sequencing analysis

The raw fastq reads underwent sequence alignment using the Spliced Transcripts Alignment to a Reference (STAR) and HOMER (http://homer.ucsd.edu/homer/ngs/rnaseq/index.html). Both raw count and TPM quantified matrix for all experiments were generated. To assess the significance of the differences observed between groups (Control, CO, SCZ), we performed nested ANOVA analysis. This choice of analysis was motivated by the fact that each patient contributed two clone samples, which served as biological replicates. Nested ANOVA allows us to account for the nested structure of the data, where clone samples are nested within donors, and to assess both between-donor variation and within-donor variation. For visualization purposes, the average TPM value of each donor's clone samples were used, but the clone factor was included in the nested ANOVA analysis. Our comparisons focused on Control vs. CO, Control vs. SCZ, and a combined group of CO & SCZ (Control vs. CO+SCZ). This approach ensures that our analysis appropriately considers the relationship between clone samples within the same donor, which is crucial in studies with such a design.

Supplementary Results

Table S1. Differential expression statistics of DLPFC of healthy rhesus macaques treated with Haloperidol low dosage (0.14 mg/kg/day) vs. placebo over six months [39] as presented in Schulmann et al. [61]

Ensembl Gene ID	Associated Gene Name	Human Ensembl Gene ID	Homology Type	logFC	t-stat.	p-value
**ENSMMUG00000016122**	PDE4A	ENSG00000065989	ortholog_one2one	-0.18	-2.04	0.049

Table S2. Linear model using age, pH and PMI as covariates t-statistic values and associated p-values for each of the brain samples datasets for PDE4 genes. T-statistic values higher than zero are marked red and t-statistic values lower than zero are marked blue. The color intensities are proportional to the t-statistic values. The q-value (corrected p-value) associated with each t-statistic value appears in the row below the t-statistic value. The q-values were calculated for each gene separately using the Benjamini- Hochberg method [92].

#	Dataset	PDE4A	PDE4B	PDE4C	PDE4D	Covariates included
1	Iwamoto 2004 t-stat	-1.80	0.82			Age, pH, PMI
	q-value	0.23	0.70			
2	Maycox 2009 t-stat	0.53	-0.15		2.11	Age, pH, PMI
	q-value	0.86	0.89		0.18	
3	Barnes 2011 t-stat	-0.85	-0.25		-0.12	Age, pH, PMI
	q-value	0.67	0.89		0.91	
4	Pietersen 2014 pyram. t-stat	0.18	0.28	-1.39	-1.70	Age, pH, PMI
	q-value	0.91	0.89	0.37	0.19	
5	Pietersen 2014 Parvalb. t-stat	-0.11	-0.20	-0.50	-0.26	Age, PMI
	q-value	0.91	0.89	0.65	0.90	
6	Chen 2013 t-stat	-1.84	-3.24	-1.89	-0.39	Age, pH, PMI
	q-value	0.23	0.02	0.30	0.90	
7	Stanley #6 t-stat	2.92	-2.38		-4.10	Age, pH, PMI
	q-value	0.10	0.15		0.01	
8	Paz 2006 t-stat	-0.29	-1.21		0.52	Age, PMI
	q-value	0.91	0.65		0.90	
9	Ramaker 2017 AnCg t-stat	-0.97	-0.87	-0.46	-1.61	Age, pH, PMI
	q-value	0.67	0.70	0.65	0.20	
10	CMC t-stat	-1.99	-1.12	-1.22	1.68	Age, pH, PMI
	q-value	0.23	0.65	0.37	0.19	
	Mean t-statistic	-0.42	-0.83	-1.09	-0.43	

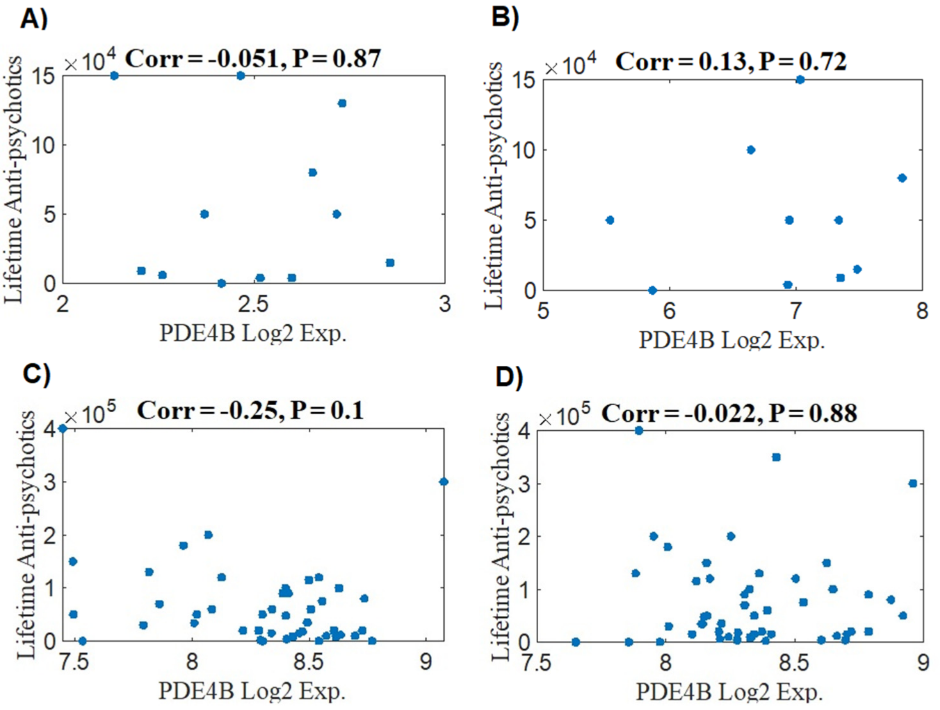

Figure S1. Pearson Correlation between antipsychotic treatment and PDE4B expression levels in the Iwamoto 2004, Stanley#6, Chen 2013 cerebellum and Parietal cortex datasets. A) Iwamoto 2004 dataset: Scatter plot of lifetime quantity of Fluphenazine or an equivalent antipsychotic (in mg) vs. gene expression, along the 12 individuals with schizophrenia for which this information was available. Each point represents one of the 12 individuals with schizophrenia. Pearson correlation and the

associated p-value are written in the title. B) The same for Stanley#6 dataset. C) The same for Chen 2013 cerebellum dataset. D) The same for Chen 2013 Parietal cortex dataset.

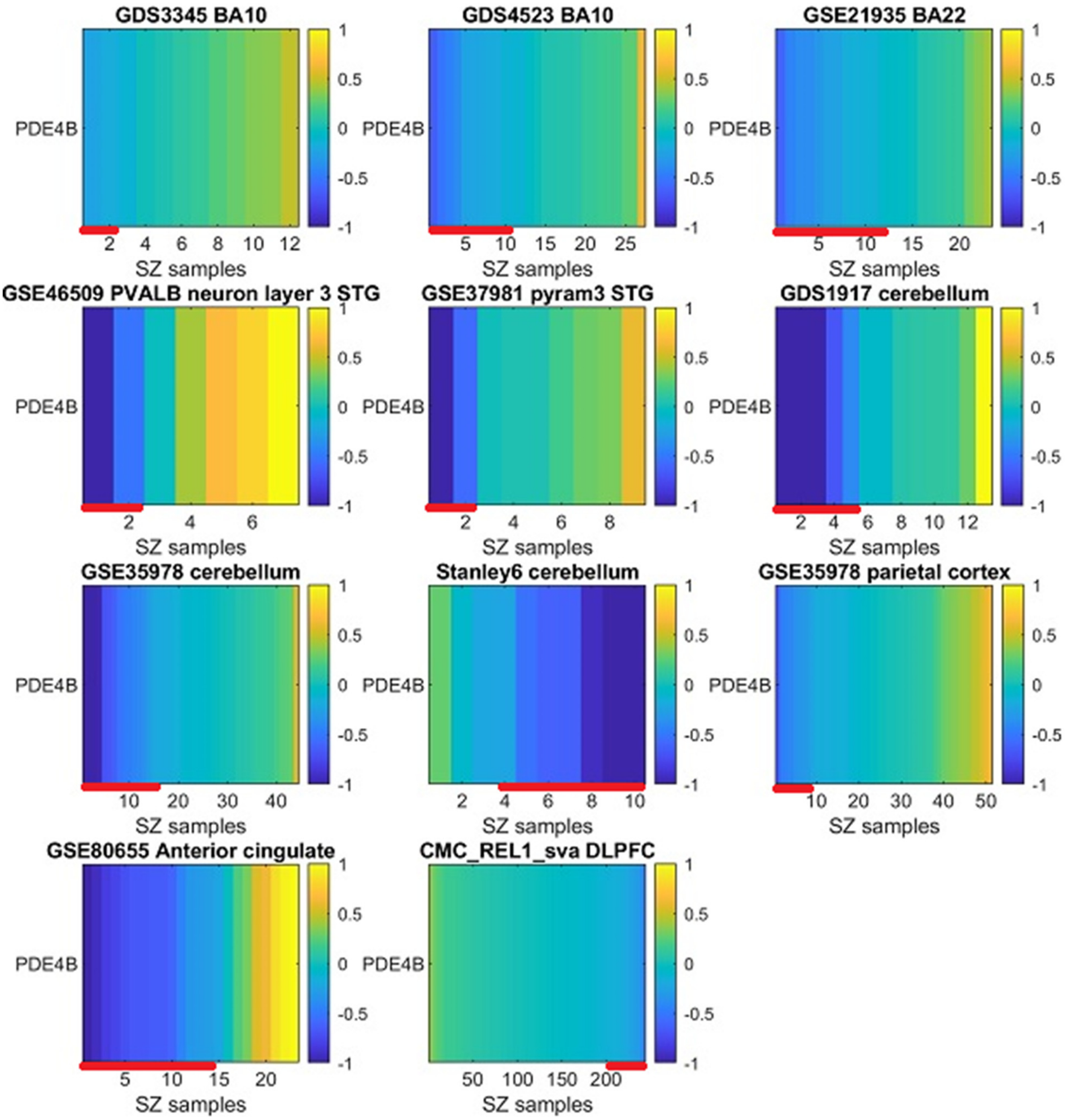

Figure S2. Per-sample Log fold change analysis. Each subplot represents one dataset. In each subplot, each column represents an individual with schizophrenia. The color in entry (j) represents the Log2 fold change of the expression of PDE4B in the individual in column j, Ej (Log2 (Ej/mean expression of the control samples)). Samples in which PDE4B is downregulated (bluish color) are marked with a red line along the x-axis.

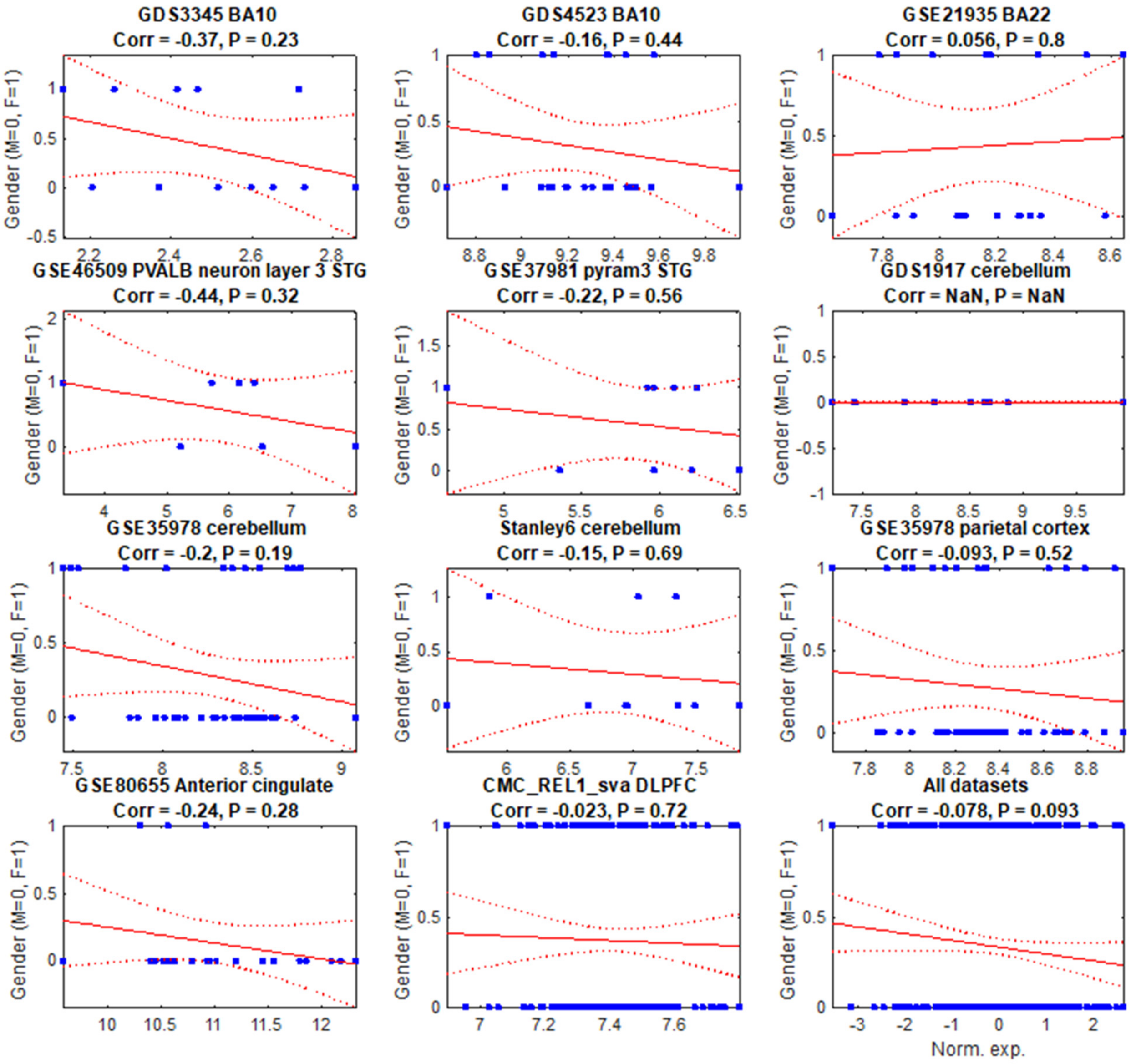

Figure S3. Pearson Correlation between PDE4B Log2 expression and sex (M=0, F=1) among the schizophrenia samples of the 11 brain samples datasets included in the meta-analysis. Each subplot represents one dataset, whose name appears in the title. The x-axis represents PDE4B Log2 expression and the y-axis represents the sex of the patients (M=0, F=1). Each blue dot represents one patient with schizophrenia. The Pearson correlation coefficients and the associated p-values appear in the titles of the subplots. The bottom right subplot represents the unified data of all datasets, where the expression was normalized in each dataset separately to have mean = 0 and standard deviation = 1.

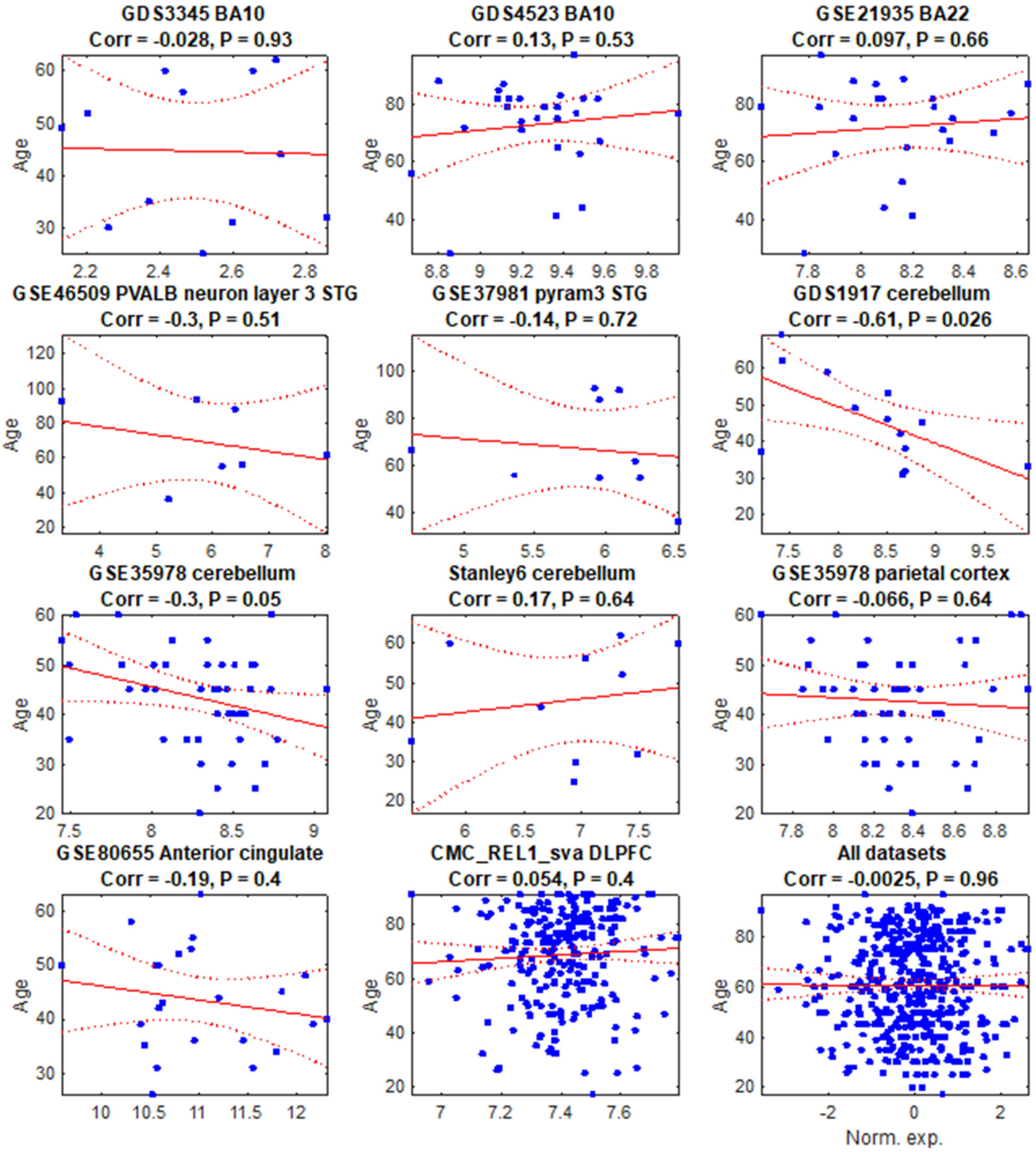

Figure S4. Pearson Correlation between PDE4B Log2 expression and the age of the patients with schizophrenia in the 11 brain samples datasets included in the meta-analysis. Each subplot represents one dataset, whose name appears in the title. The x-axis represents PDE4B Log2 expression and the y-axis represents the age of the patients. Each blue dot represents one patient with schizophrenia. The Pearson correlation coefficients and the associated p-values appear in the titles of the subplots. The bottom right subplot represents the unified data of all datasets, where the expression was normalized in each dataset separately to have mean = 0 and standard deviation = 1.

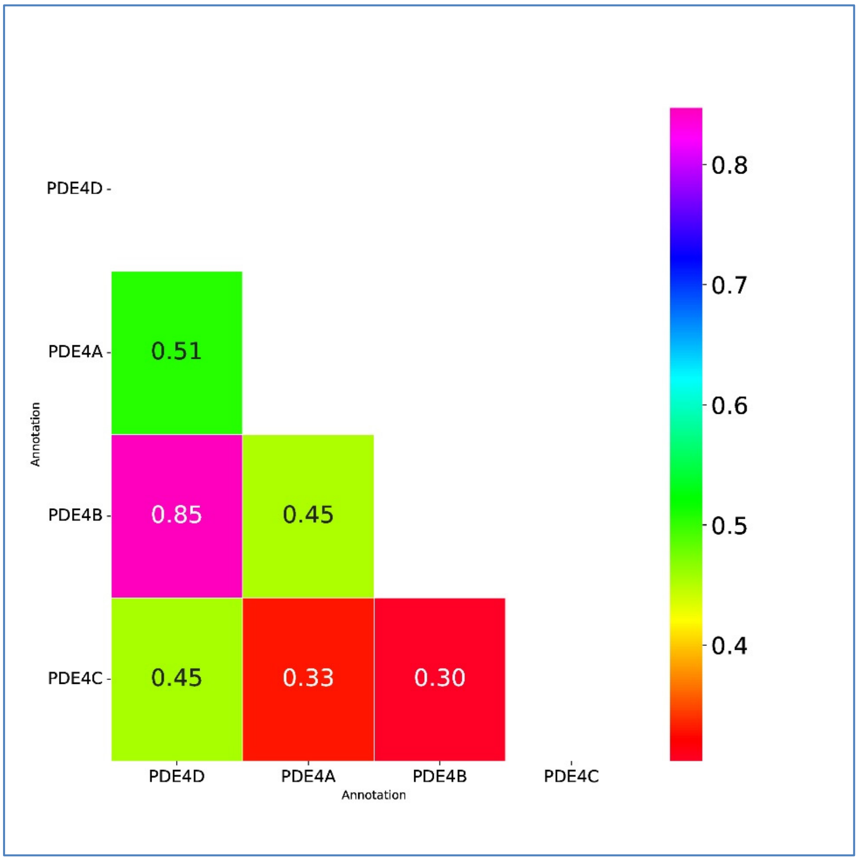

Figure S5. Inter-gene correlation between PDE4 genes. The correlation was calculated based on the average TPM matrix for all samples. The gene PDE4B was most correlated with the gene PDE4D. Overall, the expression of the four PDE4 genes was correlated, and these genes tend to be expressed together.

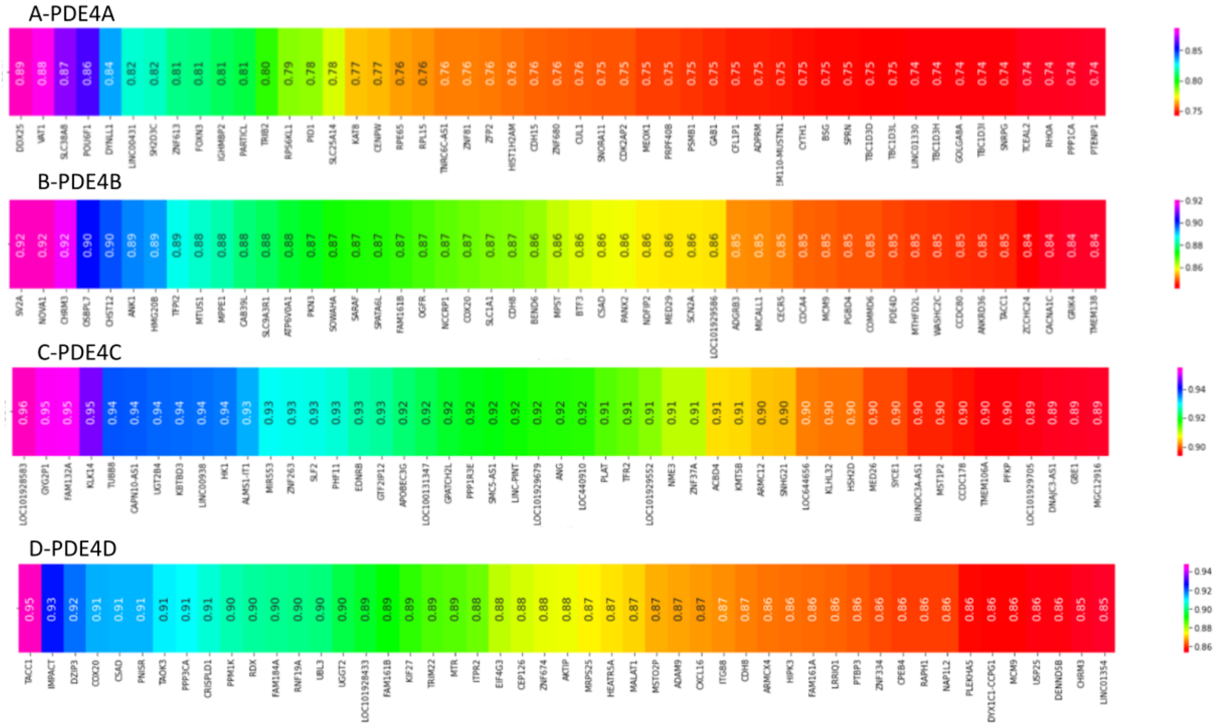

Figure S6. Correlation of other genes with PDE4 genes. The correlation was calculated based on the TPM matrix after reducing genes with low values (total TPM values, per gene, < 1.0) , this reduced the number of genes from 27k to 18k. Then, the Pearson correlation was calculated for the entire "reduced" TPM matrix, and we only took the PDE genes columns. The plot shows the top 50 correlated genes for each of the PDE4 genes (A - PDE4A, B - PDE4B, C – PDE4C, and D – PDE4D).

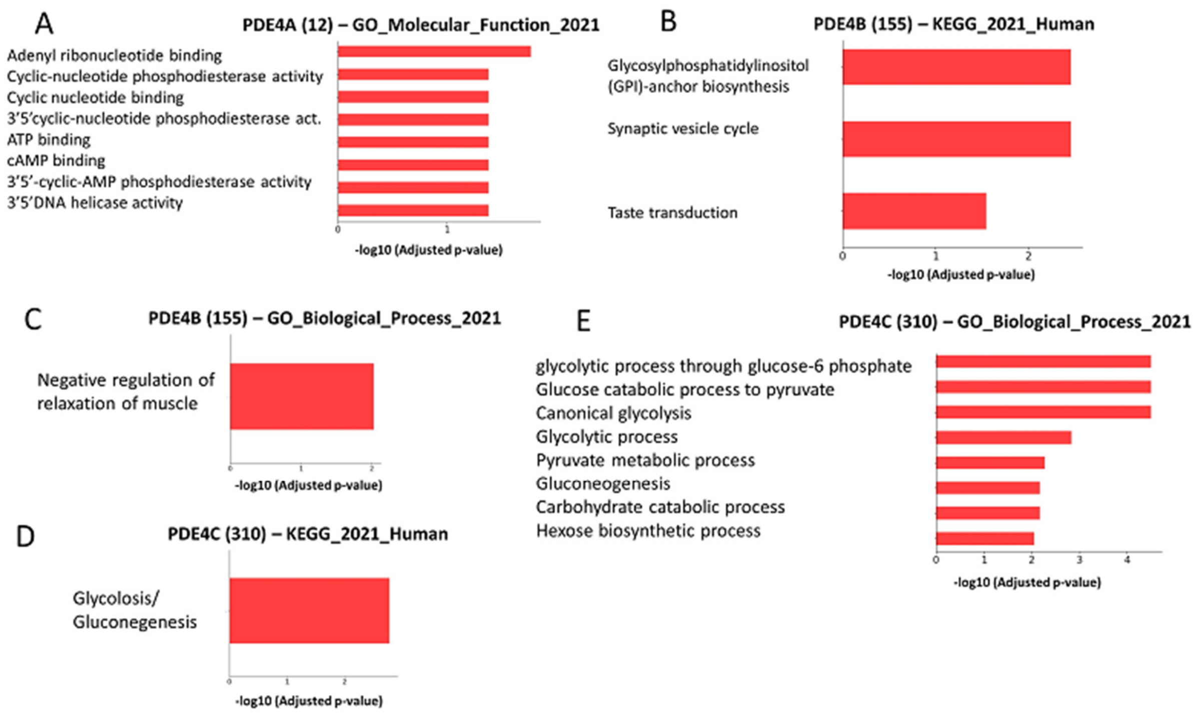

Figure S7. Pathway analysis for PDE4 correlated genes. A. The GO Molecular Function pathways enriched for genes that were correlated with the PDE4A gene. B. The KEGG pathways enriched for genes with TPM values that were correlated with the PDE4B gene. C. The GO Biological Process pathways enriched for genes with TPM that were correlated with the PDE4B gene. D. The KEGG pathways enriched for genes with TMP values that were correlated with the PDE4C gene. E. The GO Biological Process pathways enriched for genes with TMP values that were correlated with the PDE4C gene.

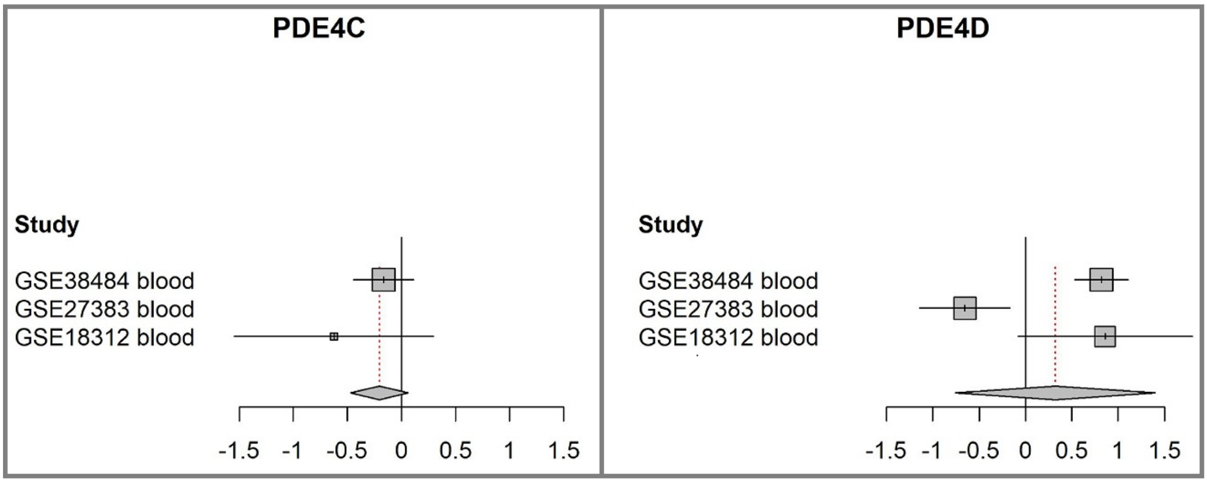

Figure S8. Meta-analysis of PDE4C and PDE4D differential expression in three datasets of blood samples of individuals with schizophrenia vs. healthy controls. Forest plot was generated using the function “forest” from the “meta” package in R, version 4.9-2 (General Package for Meta-Analysis) (Schwarzer et al., 2015). Each square represents the standardized difference (Hedges’ g (Hedges, 1981))between schizophrenia and control for a specific dataset, with the area of the square reflecting the weight (determined by the sample size) given to that dataset in the meta-analysis. Each horizontal line represents the 95% confidence interval for the mean difference.

Proxl ::MAP2::NeuN::DAPI

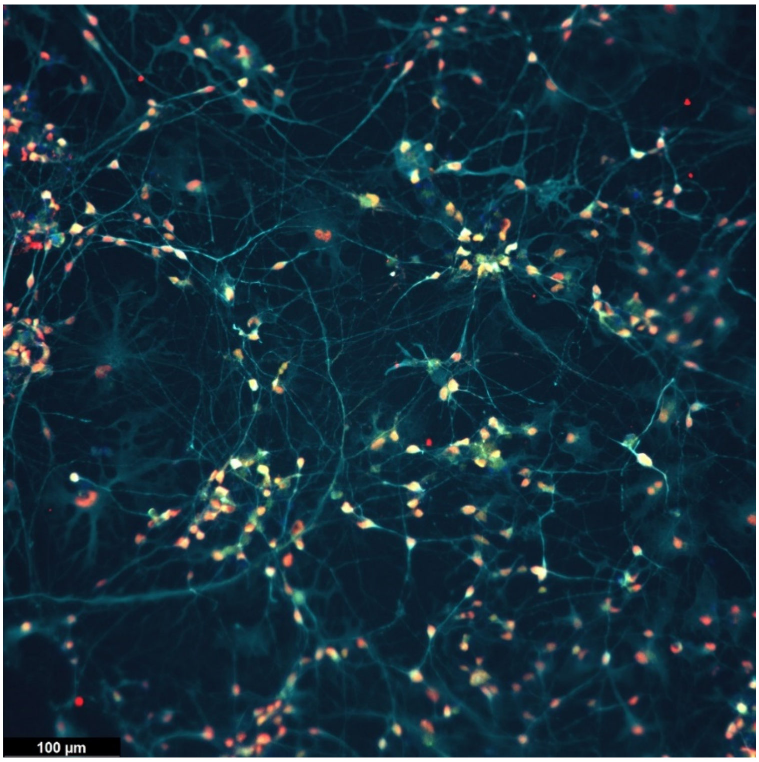

Supplementary Figure S9. ICC for Prox1, NeuN, Map2, DAPI in DG neurons. A representative image for ICC performed on a DG neuronal culture showing that a high percentage of Prox1 positive neurons.

Association between drugs' adverse effects and symptoms resembling schizophrenia

We identified 24 pharmaceutical agents with activity on PDE4B using a publicly available data source (https://go.drugbank.com/bio_entities/BE0000487). Out of 24 agents, two are not inhibitors and are thus removed. For another eight, we could not find publicly available information. Two other agents are applied topically or do not penetrate the CNS. Out of the remaining 12, none has been found to cause side effects resembling the negative or positive symptoms of schizophrenia. (Table 2S)

Table S3. Pharmacological agents with interaction with PDE4B and their related properties. The list has been accessed through: https://go.drugbank.com/bio_entities/BE0000487 Status: based on the information in the link. A= Approved, E = Experimental, I = Investigational, CNS = Central nervous system. Activity, Effect, and CNS activity were based on the information in the websites and other peer-reviewed scientific papers.

DRUG NAME	STATUS	ACTIVITY	EFFECT	CNS ACTIVITY	REFERENCE
Dyphylline	A	Inhibitor	No	V	https://reference.medscape.com/drug/lufyllin-dyphylline-343459#10
Enprofylline	A, E	Inhibitor	No	V	https://www.sciencedirect.com/topics/medicine-and-dentistry/enprofylline
Papaverine	A, E	Inhibitor	No	x	https://www.rxlist.com/papaverine-drug.htm#warnings
Caffeine	A	Inhibitor	No	V	https://ps.psychiatryonline.org/doi/10.1176/ps.49.11.1415?url_ver=Z39.88-2003&rfr_id=ori:rid:crossref.org&rfr_dat=cr_pub%20%200pubmed , https://www.ncbi.nlm.nih.gov/books/NBK519490/
Theophylline	A	Inhibitor	No	V	https://www.ncbi.nlm.nih.gov/books/NBK519024/#article-30036.s5
Daxalipram	E	Inhibitor	No info	V	
Rolipram	I	Inhibitor	No	V	Has antipsychotic properties .https://www.ncbi.nlm.nih.gov/pmc/articles/PMC6741679/
(R)-Rolipram	E	Inhibitor	No	V	
(S)-Rolipram	E	Inhibitor	No	V	
3,5-Dimethyl-1-(3-Nitrophenyl)-1h-Pyrazole-4-Carboxylic Acid Ethyl Ester	E		No info		
Filaminast	E	Inhibitor	No info	V	https://go.drugbank.com/drugs/DB02660
8-Bromo-Adenosine-5'-Monophosphate	E	Inhibitor	No info	V	https://pubmed.ncbi.nlm.nih.gov/10385419/
1-(2-Chlorophenyl)-3,5-Dimethyl-1h-Pyrazole-4-Carboxylic Acid Ethyl Ester	E		No info	V	https://go.drugbank.com/drugs/DB03807
Cilomilast	I	Inhibitor	No	V	https://www.tandfonline.com/doi/abs/10.1517/13543784.10.7.1361
S,S-(2-Hydroxyethyl)Thiocysteine	E		No info	X	https://go.drugbank.com/drugs/DB04530
Roflumilast	A	inhibitor	loss of interest or pleasure + trouble concentrating	V	1. https://www.sciencedirect.com/science/article/pii/S0166432816300262?via%3Dihub 2. https://www.drugs.com/sfx/roflumilast-side-effects.html
Crisaborole	A, I	Inhibitor	Irrelevant - Topical Medication		
Piclamilast	E	Inhibitor	No	V	https://go.drugbank.com/drugs/DB01791
Iloprost	A, E	Inhibitor	No	V	1. https://medlineplus.gov/druginfo/meds/a612032.html#side-effects 2. https://go.drugbank.com/drugs/DB01088
1-ethyl-N-(phenylmethyl)-4-(tetrahydro-2H-pyran-4-ylamino)-1H-pyrazolo[3,4-b]pyridine-5-carboxamide	E		No info		
4-[8-(3-nitrophenyl)-1,7-naphthyridin-6-yl]benzoic acid	E	Inhibitor	No info		https://jpet.aspetjournals.org/content/301/1/241.full
Ibudilast	I	Inhibitor	Depression	V	1. https://www.nejm.org/doi/full/10.1056/NEJMoa1803583 2. https://www.ncbi.nlm.nih.gov/pmc/articles/PMC2675769/
Theobromine	I	Inhibitor	Negative mood effects at high doses	V	https://www.ncbi.nlm.nih.gov/pmc/articles/PMC3672386/

References

90. Oldham, M.C.; Konopka, G.; Iwamoto, K.; Langfelder, P.; Kato, T.; Horvath, S.; Geschwind, D.H. Functional Organization of the Transcrip-tome in Human Brain. Nature Neuroscience 2008 11:11 2008, 11, 1271–1282, doi:10.1038/nn.2207.

91. Andrews, D.F. A Robust Method for Multiple Linear Regression. Technometrics 1974, 16, 523–531, doi:10.1080/00401706.1974.10489233.

92. Benjamini, Y.; Hochberg, Y. Controlling the False Discovery Rate: A Practical and Powerful Approach to Multiple Testing. Journal of the Royal Statistical Society. Series B (Methodological) 1995, 57, 289–300.
